# Electroencephalographic Recording of the Movement-Related Cortical Potential in Ecologically Valid Movements: A Scoping Review

**DOI:** 10.3389/fnins.2021.721387

**Published:** 2021-09-28

**Authors:** Sharon Olsen, Gemma Alder, Mitra Williams, Seth Chambers, Mads Jochumsen, Nada Signal, Usman Rashid, Imran Khan Niazi, Denise Taylor

**Affiliations:** ^1^Rehabilitation Innovation Centre, Health and Rehabilitation Research Institute, Auckland University of Technology, Auckland, New Zealand; ^2^Department of Health Science and Technology, Aalborg University, Aalborg, Denmark; ^3^Centre for Chiropractic Research, New Zealand College of Chiropractic, Auckland, New Zealand

**Keywords:** movement related cortical potential (MRCP), electroencephalograph (EEG), ecological validity, review (article), rehabilitation, movement, bereitschaftspotential (BP), contingent negative variation (CNV)

## Abstract

The movement-related cortical potential (MRCP) is a brain signal that can be recorded using surface electroencephalography (EEG) and represents the cortical processes involved in movement preparation. The MRCP has been widely researched in simple, single-joint movements, however, these movements often lack ecological validity. Ecological validity refers to the generalizability of the findings to real-world situations, such as neurological rehabilitation. This scoping review aimed to synthesize the research evidence investigating the MRCP in ecologically valid movement tasks. A search of six electronic databases identified 102 studies that investigated the MRCP during multi-joint movements; 59 of these studies investigated ecologically valid movement tasks and were included in the review. The included studies investigated 15 different movement tasks that were applicable to everyday situations, but these were largely carried out in healthy populations. The synthesized findings suggest that the recording and analysis of MRCP signals is possible in ecologically valid movements, however the characteristics of the signal appear to vary across different movement tasks (i.e., those with greater complexity, increased cognitive load, or a secondary motor task) and different populations (i.e., expert performers, people with Parkinson’s Disease, and older adults). The scarcity of research in clinical populations highlights the need for further research in people with neurological and age-related conditions to progress our understanding of the MRCPs characteristics and to determine its potential as a measure of neurological recovery and intervention efficacy. MRCP-based neuromodulatory interventions applied during ecologically valid movements were only represented in one study in this review as these have been largely delivered during simple joint movements. No studies were identified that used ecologically valid movements to control BCI-driven external devices; this may reflect the technical challenges associated with accurately classifying functional movements from MRCPs. Future research investigating MRCP-based interventions should use movement tasks that are functionally relevant to everyday situations. This will facilitate the application of this knowledge into the rehabilitation setting.

## Introduction

The movement-related cortical potential (MRCP) is an event-related potential that can be recorded over various centroparietal brain regions prior to, and at the onset of, voluntary movement (Shibasaki and Hallett, 2006). It reflects motor planning and is detectable in self-paced, cued, and imagined movement (Deeke, 1996). In self-paced movement the MRCP is commonly referred to as the bereitschaftspotential or readiness potential, while in cued movement it is termed the contingent negative variation (Shibasaki and Hallett, 2006; Shakeel et al., 2015). For the purposes of this review, the umbrella term MRCP will be utilized.

**FIGURE 1:**
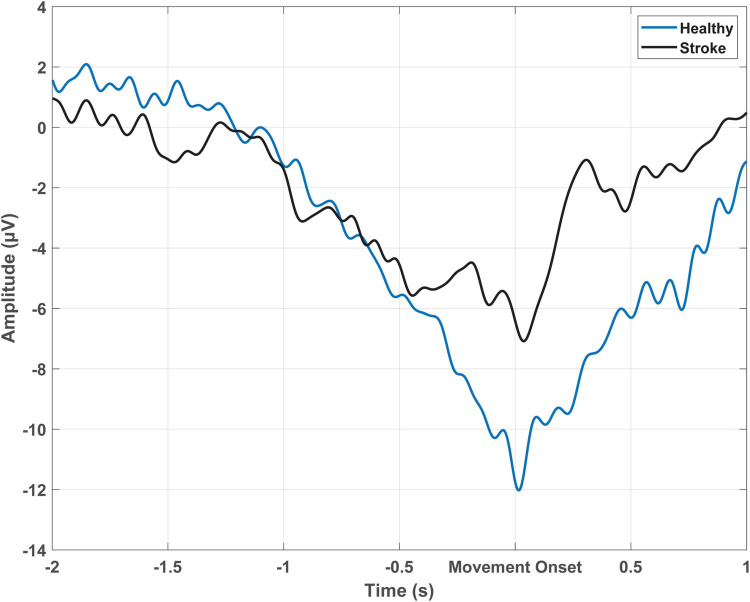
Epoch-averaged MRCP from Cz which has been filtered with a large Laplacian filter for one participant with stroke (51 trials) and one healthy participant (54 trials) performing cued voluntary ballistic ankle dorsiflexion.

The MRCP is commonly recorded using surface electroencephalography (EEG), where electrodes placed on the scalp measure voltage changes correlating with underlying activity in the superficial layers of the cortex (Holmes and Khazipov, 2007; Kirschstein and Kohling, 2009). A typical MRCP begins with a slow negative shift around 1.5–2 s prior to movement onset, with peak negativity observed around the time of movement onset, followed by a positive shift after movement execution (Do Nascimento et al., 2006; Shibasaki and Hallett, 2006; Shakeel et al., 2015) (refer to Figure 1). The primary generators of the MRCP are thought to be the bilateral supplementary motor areas, bilateral pre-supplementary motor areas, bilateral cingulate motor areas, and the contralateral M1, with some evidence also suggesting involvement of the ipsilateral M1 (Mackinnon, 2003). The MRCP is easily detected over the central electrodes near the midline. For finger movements the MRCP amplitude is largest at the C1 or C2 electrodes (International 10–20 system) (Shibasaki et al., 1980), whereas for ankle movements the amplitude peaks at the Cz or CPz electrode (Do Nascimento et al., 2006). The timing and amplitude of the MRCP varies with the type of movement, preparatory state (cued or self-paced), speed of the task, force required, the level of uncertainty about the type of movement, and the presence of neurological conditions (Brunia, 2003; Ikeda and Shibasaki, 2003; Rektor, 2003; Do Nascimento et al., 2006).

MRCP signals have been investigated across different fields such as biomedical engineering, neurophysiology, and clinical research. Observational research, where MRCP data is recorded in a sample of participants under various task-related or environmental conditions, has informed our understanding of the phases and characteristics of the MRCP, dating back to its discovery in 1964 (Kornhuber and Deecke, 1964; Kornhuber and Deecke, 1965). While these observational studies have largely focused on the MRCP during simple, single-joint movements (Jahanshahi and Hallett, 2003), there is a developing body of research examining the MRCP signal during more complex tasks (Bibian et al., 2017; Eilbeigi and Setarehdan, 2018b). MRCP research has also focused on the development of online and offline brain–computer interfaces (BCIs) (Mrachacz-Kersting et al., 2012; Niazi et al., 2012; Xu et al., 2014; Jochumsen et al., 2015a). Accurate recording and processing of MRCPs by BCIs may allow users to control assistive devices, such as BCI-triggered lower limb exoskeletons (Lopez-Larraz et al., 2016) or upper limb neuroprostheses (Müller-Putz et al., 2019; Ofner et al., 2019). In addition, BCIs can deliver MRCP-based neuromodulatory interventions. These interventions have been investigated in experimental studies, where the researcher manipulates the delivery of the intervention and measures the effects on outcomes of interest. Studies have shown that MRCP-based neuromodulatory interventions induce neuroplasticity and improve lower limb impairment following stroke (Mrachacz-Kersting et al., 2016, 2019b; Olsen et al., 2020). A further area of MRCP research concerns the effects of motor learning on changes in MRCP onset and amplitude (Hatta et al., 2009; Wright et al., 2012a; Berchicci et al., 2017); in this case, the MRCP is used as the outcome measure within an experimental study. For example, a decrease in MRCP amplitude has been observed following repeated motor task training (Jochumsen et al., 2017) which may reflect lower cortical effort required to complete the task (Wright et al., 2011).

Research exploring the MRCP has particular relevance to the field of neurological rehabilitation through the enhanced understanding of: motor impairment, recovery processes following neurological injury, and the effect of rehabilitation interventions. However, a key limitation of its application to rehabilitation is the aforementioned focus on understanding the MRCP during simple, single-joint movements, such as isolated finger flexion or ankle movements (Wright et al., 2011; Ahmadian et al., 2013). These tasks bear little resemblance to movements people perform in real life or in rehabilitation, and therefore lack ecological validity. Ecological validity refers to the extent to which a movement being analyzed for research purposes resembles actual human behavior carried out in real-world environments (Davids, 1988). There are many examples of limited ecological validity in the field of MRCP research. MRCPs recorded during simple single-joint movements have been used to determine differences between experts and novices, yet the real-world task under investigation was much more complex (e.g., rifle shooting, martial arts) (Kita et al., 2001; Di Russo et al., 2005; Hatta et al., 2009). The effect of task training on healthy participants has been assessed using the MRCP recorded during simple grasping movements, rather than the fine motor task that was trained (Jochumsen et al., 2017), and the effect of rehabilitation interventions following stroke has been assessed using a simple finger flexion task rather than the goal-directed, complex upper limb tasks being rehabilitated (Kopp et al., 1999; Tarkka et al., 2008). In addition, an MRCP-based neuromodulatory intervention has been applied during a simple ankle dorsiflexion task in people with stroke (Mrachacz-Kersting et al., 2016, 2019b; Olsen et al., 2020), yet this single-joint movement lacks specificity to the real-world tasks required for lower limb function (for example, sit to stand, walking, or climbing stairs). This lack of ecological validity in MRCP research may be attributed to challenges that arise while recording EEG during more complex movements as the low-frequency MRCP signal can be easily masked by artifacts due to background noise, eye blinks, or other body movements (Wright et al., 2011; Ahmadian et al., 2013). However, with advances in technology and knowledge, recording the MRCP under more real-world conditions has become more feasible (Jochumsen et al., 2020a; Schwarz et al., 2020a), and this progression is essential to understanding the MRCP during real-world movement tasks. Without this understanding, knowledge about the MRCP will remain limited to controlled laboratory-based paradigms, rather than the complex movement tasks that are the focus of neurological rehabilitation (Langhorne et al., 2009, 2011; Pollock et al., 2014).

Synthesis of the MRCP literature is a challenge due to its diverse research objectives and the different terminologies used across various fields of research (Jahanshahi and Hallett, 2003). Previous literature reviews have focused on the characteristics of the MRCP and their physiological implications (Shibasaki and Hallett, 2006), the application of the MRCP to motor learning (Wright et al., 2011), its use as a predictor of an upcoming movement (Ahmadian et al., 2013; Shakeel et al., 2015), and its potential to assess outcomes following stroke (Monge-Pereira et al., 2017). However, no reviews have specifically focused on understanding the MRCP during ecologically valid movements. Therefore, this paper uses a scoping review method to explore this body of literature (Munn et al., 2018). Scoping reviews provide a systematic approach to determine the volume of evidence in an area and to provide an overview of its focus (Munn et al., 2018). The aim of this scoping review is to identify, describe, and synthesize the research evidence investigating the MRCP in ecologically valid movement tasks.

## Materials and Methods

### Search Strategy

A database search was conducted to identify literature across the biomedical engineering, neurophysiology, and clinical fields of research (refer to Table 1 for search terms, latest search 9th March 2021). The databases searched were: MEDline, CINAHL, SportDISCUS, Scopus, AMED, and Web of Science. Results from all databases were exported to EndNote X9, where duplicates were subsequently removed.

**TABLE 1:** Search strategy.

	Search terms
#1	CNV OR “contingent negative variation*” OR MRCP OR “movement related cortical potential*” OR “movement-related cortical potential*” OR bereitschaftspotential* OR “readiness potential*” OR “negative slope potential*”
#2	“reach* to grasp*” OR “reach* and grasp*” OR reach* OR throw* OR threw OR pull* OR gait OR “gait initiation” OR “gait-initiation” OR ambula* OR locomot* OR “sit to stand” OR “sit-to-stand” OR “standing up” OR stepping OR stepped OR walk OR walking OR walked OR step OR “step-up” OR “step up” OR “step initiation” OR “step-initiation” OR kicking OR kicked OR kick OR jump OR jumping OR “motor performance” OR “movement performance” OR “motor learning” OR “motor training” OR “motor control” OR “movement control” OR “complex task*” OR (skilled [proximity search within 3 words] task*) OR “brain computer interface*” OR “brain-computer interface*” OR “brain-machine interface” OR “brain machine interface” OR BCI
#3	gene OR genes or genetic* OR genome* OR hereditary OR DNA OR “copy number variation*”
#4	(#1 AND #2) NOT #3

### Screening

Title and abstract screening was independently completed by two pairs of reviewers (SO and MW; GA and SC) using the inclusion/exclusion criteria described in Table 2. Results were compared and any disagreements were settled by discussion. Full-text versions of the screened articles were then independently assessed by pairs of reviewers (MW and SC; SO and GA) for inclusion in the final review. Results were compared and any disagreement or uncertainty was settled by consultation with a third reviewer (SO or IN). Additional references were sourced by hand-searching reference lists of relevant articles. The initial inclusion criteria required studies to investigate the MRCP during voluntary upper or lower limb multi-joint movement; these were defined as limb movements involving two or more segments moving either simultaneously or sequentially. Due to the large volume of articles meeting the initial inclusion criteria, the criteria were refined to focus on the ecological validity of the movement task under investigation and its generalizability to rehabilitation. The final criteria required the MRCP movement task to be categorized in the activity or participation domains of the International Classification of Functioning, Disability and Health (ICF) (World Health Organisation, 2001). Components of walking were included (e.g., stepping), but upper limb tasks that involved only part of the task (due to simplification), or that had no clear functional application, were excluded. For example, one excluded study modified a drawing task so that the forearm and wrist were fully supported by an apparatus only allowing horizontal shoulder and elbow movement, with the fingers fixed around a cone (Fang et al., 2007).

**TABLE 2:** Final inclusion and exclusion criteria.

	Inclusion	Exclusion
**Participants**	Aged over 18 years	Animal studies
**Study characteristics**	Investigation of scalp-recorded MRCP either through observation, as a measure of intervention efficacy, or as a component of an intervention, during voluntary movement classified as an activity or participation task under the ICF. Components of walking included (e.g., stepping).	Recorded MRCP during: imagined movement only, involuntary movement only, movement carried out by an exoskeleton or other robotic device, single joint movements, movements of only the hand/wrist/radioulnar joints or only the foot/ankle joints, and upper limb tasks that involved only part of the task or with no clear functional application.
**Publication**	Full text articles published in English including conference proceedings.	
**Type of research**	Primary research: randomized, non-randomized, experimental, case reports, observational.	Review articles, expert opinions or anecdotal reports.

### Data Extraction and Analysis

The following information was extracted: participant characteristics, study design, study aim, purpose of the MRCP recording, task(s) used to record the MRCP, whether movements were self-paced or cued, and the key findings related to the MRCP. In addition, information about recording methods including EEG recording sites, amplifiers, filtering, pre-processing, and epochs (time/response locking and duration) were also extracted. Data extraction was carried out by three authors (MW, SC, or SO), and checked for accuracy by additional authors (SO, MJ, and UR). Extracted data was synthesized and analyzed descriptively with a focus on common objectives, and similarities and differences between populations, types of motor tasks, and recording methods. Gaps in the literature were identified.

## Results

### Identification and Selection of Studies

**FIGURE 2:**
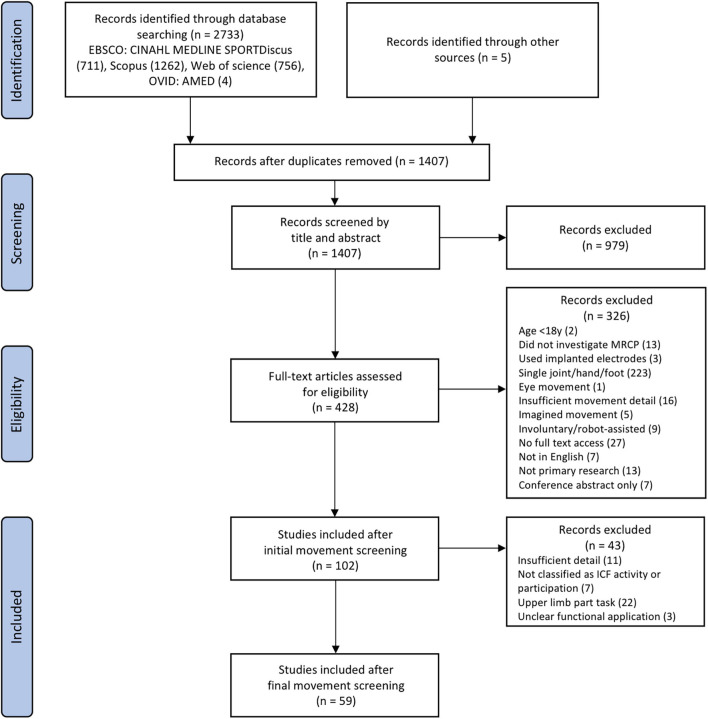
Flow chart detailing screening process.

The literature search includes studies published prior to 9th March 2021. A total of 102 articles met the initial eligibility criteria, and after further refinement of the inclusion criteria, 59 articles were finally included (refer to Figure 2).

### Description of Included Studies

Descriptive data for all included studies can be found in Table 3 for observational studies and Table 4 for experimental studies. A description of the EEG recording and processing methods for all studies can be found in Supplementary Table 1.

**TABLE 3:** Observational Studies.

Author	Study Design	Study Aim	Participants	Mode	Movement task	Key findings
**Specialized goal-directed activities**						
Berchicci et al., 2017	Between-group, cross sectional (novice vs. expert and simple vs. complex)	To investigate the effect of expertise on brain activity (the MRCP) during a bimanual coordinative task (juggling) in ecological settings	*n* =38 jugglers and novices, data included for *n* =28. Jugglers *n* =14 (2F, 32 ±6 years). Novices *n* =14 (3F, 30 ±5 years)	Self-paced	Novices: seated 1-ball and 2-ball juggling. Experts: seated 2-ball and 3-ball juggling.	– Data excluded for *n* =10 due to low signal-to-noise ratio. – Prefrontal MRCPs had earlier onsets and larger amplitudes for complex vs. simple task, for novices (2-ball vs. 1-ball) and experts (3-ball vs. 2-ball). – For the same task (2-ball), Cz MRCP amplitude (500 ms window prior to onset) was larger in experts vs. novices.
Euler et al., 2016	Single-group, cross-sectional, multiple movement conditions	To investigate the neurocognitive correlates (psychomotor speed, executive function, and working memory) of MPLs and MRCPs that were simultaneously recorded from healthy individuals during complex motor sequencing (with familiar vs. novel contexts)	*n* =47 healthy psychology students, data included for *n* =40 (26F, 26 ±9 years)	Cued	Sequences of pushing joystick, twisting joystick, and tapping button. “Familiar” context had standard visual cue to perform practiced sequence. “Novel” context had unfamiliar/complex visual cue to perform same sequence.	– Data excluded for *n* =7 due to technical failure, incorrect performance, excessive artifacts, or MRCP amplitude > 3.5 SDs of sample. – MRCP had a later PN and smaller amplitude (FCz) in the novel context. – Executive function was correlated with MRCP amplitude and PN timing for both familiar and novel contexts. Psychomotor speed was correlated with PN timing in both contexts.
Fromer et al., 2012	Single-group, cross-sectional, multiple movement conditions (simple vs. complex)	To investigate the feasibility of studying the preparatory process for complex goal-directed tool use (aim phase of throwing with no aim, large target, and small target) using the ERP method (including MRCP)	*n* =25 healthy, data included for *n* =18 (9F, 23 ±5 years)	Cued	Dart throwing using Wii remote (un-aimed, large target aimed, small target aimed) vs. simple button release on Wii remote.	– Data excluded for *n* =7 due to poor data quality. – MRCP amplitude was largest in small target dart throwing (most difficult), followed by large target dart throwing and un-aimed throwing, and smallest in simple button release.
Jung, 1982	Single-group, cross-sectional, multiple movement conditions	To investigate the programming and steering of voluntary goal-directed action in man by simultaneous recordings of brain potentials (MRCP) and eye and muscle activity before and during movement	*n* =10 healthy males (age not reported)	Self-paced and cued	Rapid boxing jabs toward target and slow forefinger pointing toward target.	– Descriptive analysis only. Duration of MRCP longer for rapid punch (1–1.5 s) vs. slow pointing (1.5–2 s).
Mann et al., 2011	Between-group, cross-sectional (novice vs. expert)	To investigate whether modulations of the quiet eye period and BP discriminate expertise and performance differences while expert and near-expert golfers performed the golf putt	*n* =20 golfers mExperts *n* =10 (26 ±7 years). Near-expert *n* =10 (26 ±6 years). Gender not reported.	Self-paced	Golf putting	– MRCP amplitude larger in expert group vs. near-expert group. – MRCP amplitude did not influence accuracy. – As quiet eye period increased, so did MRCP amplitudes.
Martinez-Exposito et al., 2017	Single-group, cross-sectional, multiple movement conditions (simple vs. complex)	To investigate CNV and ERD patterns when subjects perform four different kinds of tasks involving the UL or LL and analytic (single joint) and coordinated actions (multiple joints)	*n* =7 healthy (3F, age range 23–30 years)	Cued	UL: reaching to touch target at 75 cm (complex) and shoulder flexion (simple). LL: 2 pedaling cycles (complex) and knee extension (simple).	– No differences for UL simple vs. complex, or LL simple vs. complex. – LL complex task had larger PN amplitude than UL complex task. – LL simple task had larger PN amplitude than UL simple task.
Nann et al., 2019	Single-group, cross-sectional, multiple movement conditions	To investigate the impact of possible life-threatening decision making on the BPs spatiotemporal dynamics	*n* =2 semi-professional male cliff divers (19 years)	Self-paced	192-meter bungee jump vs. jump off 1-meter platform, 12–16 jumps for each condition.	– No significant differences in MRCP onset and amplitude in 192-meter bungy jump vs. 1-meter jump.
O’connor, 1986	Between-group, cross-sectional, multiple movement conditions	To investigate whether a change in background motor set (the motor task performed after smoke inhalation) would alter the smoker’s smoking pattern and whether smoking (vs. sham) would principally affect extroverts general motor preparation (MRCP during tapping task)	*n* =10 smokers Introverts *n* =5 (mean 29 years) Extroverts *n* =5 (mean 27 years) Gender matched	Self-paced	Lifting cigarette to mouth and inhaling. This was performed prior to three different conditions: resting, finger tapping, and a moving a ring over a wire without the two touching.	– Smoking-locked MRCP amplitudes (RP) were larger for introverts vs. extroverts. – After smoking, tapping-locked MRCP amplitudes (RP) were larger for extroverts. – After smoking, tapping performance improved for introverts only.
Skrzeba and Vogt, 2018	Between-group, cross-sectional (novice vs. expert)	To investigate central neuronal motor behavioral processes (MRCPs) preceding the short badminton backhand serve with the non-dominant and dominant hand (of expert and novice players)	*n* =16 male badminton players Experts *n* =8 (26 ±5 years) Novices *n* =8 (22 ±4 years)	Self-paced	Backhand badminton serve with dominant and non-dominant hands	– PN of MRCP was larger in expert’s dominant hand compared to novice’s dominant hand. No significant differences between the non-dominant hands.
Tomyta and Seki, 2020	Single-group, cross-sectional, multiple movement conditions	To investigate the effects of tapping style on motor performance and neural activity in self-paced and synchronization tapping tasks in three conditions (drum sticking, 1-finger tapping, and 4-finger tapping)	*n* =12 healthy right-handed non-musicians (5F, 20 ±0.9 years)	Self-paced and cued	Drum-stick tapping, index finger keyboard tapping, and 4-finger keyboard tapping, in self-paced and cued conditions (auditory stimuli 70 beats/min).	– FC1 pre-movement negativity, similar to MRCP, was significantly larger in 4-finger tapping than drum-stick and 1-finger tapping, but not different between cued and self-paced conditions (120–1 ms window). – Significantly larger post-movement positive peak in drum-stick tapping vs. 4-finger tapping (80–220 ms window).
Vogt et al., 2017	Between-group, cross-sectional (novice vs. expert)	To investigate differences of central neuronal motor behavior between skilled and less skilled archery novices during real sport-specific movements	*n* =16 healthy males (30 ±6 years) without archery experience, divided into skilled vs. less-skilled.	Self-paced	Archery: releasing an arrow toward a target 15 m away	–MRCP onset was later and amplitude larger (RP) in skilled vs. less skilled participants.
Wright et al., 2012b	Between-group, cross-sectional (novice vs. expert)	To investigate MRCP differences in experienced guitarists and non-musicians using an ecologically valid guitar-playing task	*n* =20. Experienced guitarists *n* =10 (0F, 37 ±14 years). Non-musicians *n* =10 (5F, 24 ±7 years).	Self-paced	Guitar playing (G major scale)	– MRCP negative slope (steeper phase of increased negativity prior to PN of MRCP) was earlier and of larger amplitude in non-musicians vs. experienced guitarists. – PN of MRCP was larger in non-musicians vs. experienced guitarists.
**Walking-related tasks**						
Berchicci et al., 2020	Single-group, cross-sectional, multiple movement conditions	To investigate the neural correlates of forward- and backward-oriented stepping by means of MRCP data	*n* =13 healthy (6F, 22 ±3.9 years)	Self-paced	Stepping both feet forward onto force platform, then stepping both feet backward.	– Data excluded for *n* =2 due to recording issues – MRCP amplitude significantly larger for backward stepping vs. forward stepping – Greater prefrontal and frontal activity during early MRCP (−1.5t o −0.5 s) in backward stepping.
Bodda et al., 2020	Single-group, cross-sectional, one movement condition	To investigate cortical activity (including the MRCP) during the stance and swing phases of the gait cycle.	*n* =10 healthy (18–23 years)	Cued	Walking (8 steps)	– Alternating positive and negative potentials observed (F3). Negative peaks corresponded to contralateral heel strike. Positive peaks corresponded to contralateral push off. See note #
Braquet et al., 2020	Single-group, cross-sectional, multiple movement conditions	To investigate whether attentional load modification can modulate cortical activation during GI through the analysis of response-locked ERPs and ERSPs (including the MRCP)	*n* =30 healthy (16F, 39 ±14 years)	Cued	Forward step (with left or right foot) preceded by warning signal (no cue, or star placed center, left or right of screen) then cue to step (simple or disorientating arrows toward left or right).	– No significant differences in MRCP amplitude or latency between different warning signals and cues to step (differing levels of attentional load).
Do Nascimento et al., 2005	Single-group, cross-sectional, multiple movement conditions	To investigate whether MRCPs are influenced by variations in direction of stepping and GI	*n* =8 healthy (4F, 24 ±4 years)	Self-paced	Forward and backward GI consisting of 3 steps. Forward, and backward single steps. One lateral single step.	– Location and amplitude of MRCP varied between GI in different directions (forward, backward, lateral) and between stepping in different directions, with backward tasks having the largest MRCP amplitude. – Gait tasks were mainly differentiated in the early MRCP; stepping tasks were differentiated in later MRCP.
Fearon et al., 2021	Between-group, cross-sectional, multiple movement conditions	To investigate the behavioral impact of stepping-in-place on a simple response time task and the underlying electrophysiological markers for decision-making, response conflict and motor preparation (MRCP)	*n* =10 PD with freezing of gait (FOG), data included for *n* =8 (1F, 65 ±7 years) *n* =10 PD without FOG (6F, 63 ±8 years) *n* =7 healthy (3F, 25 ±5 years)	Cued	Pressing button on Wii remote while either: – Sitting (single task) – Walking in place with walking frame (dual task)	– Data excluded for *n* =2 due to technical errors – MRCP amplitude larger for people with PD and FOG compared to people with PD without FOG and healthy. – MRCP amplitude not significantly different between single-task and dual-task conditions, however MRCP duration longer and response time slower under dual-task condition in people with PD and FOG.
Jeong et al., 2017	Single-group, cross-sectional, multiple movement conditions, multiple measurement or processing conditions	To investigate a single-trial RP (MRCP) detection system for control of a lower-limb exoskeleton	*n* =5 healthy (2F, 26–29 years)	Self-paced	Voluntary half-step walking (with and without an exoskeleton).	– Average single trial detection accuracy of stepping in exoskeleton was 76.7% with CAR filter and 80.7% with Laplacian filter.
Jiang et al., 2015	Single-group, cross-sectional, multiple movement conditions	To investigate the detection of the intention of GI from MRCPs	*n* =9 healthy (3F, 21–38 years)	Self-paced	Forward step then backward step to return foot to start position.	– TPR for detecting MRCP onset was 76.9 ±8.97%, while FPR was 2.93 ±1.09 per min. – Average detection latency of PN of MRCP was −180 ± 354 ms.
Jochumsen and Niazi, 2020b	Single-group, cross-sectional, multiple movement conditions	To detect and classify six different movement tasks (using the MRCP) of the lower extremities that are used in activities of daily living	*n* =13 healthy (5F, mean 24 years)	Cued	Stand-to-sit, sit-to-stand, GI, step-up, side-step, backward step.	– GI had smaller MRCP amplitude compared to step-up, side-step, backward step, and stand-to-sit. – Stand-to-sit had larger MRCP amplitude than other tasks. – 54 ±3% of all movement types were classified correctly. Highest classification accuracies were obtained for stand-to sit and sit-to-stand (71 ±6% and 67 ±5%), whereas step-ups and backward steps had the lowest classification accuracies (36 ±5% and 42 ±6%).
Karimi and Jiang, 2019	Single-group, cross-sectional, multiple movement conditions (simple vs. complex), multiple measurement or processing conditions	To investigate the performance of a semi-blind source extraction algorithm (reference-based source extraction (RBSE)) to extract the MRCP during GI	*n* =5 healthy (age/gender not reported)	Cued	Forward step and seated DF	– When algorithms were trained with only ankle DF data, RBSE method had the highest performance index quantifying the signal-to-noise ratio (2.43 ±1.23). – When algorithms were trained with ankle DF and stepping data, common spatial pattern method had highest performance index (2.60 ± 1.04), and RBSE method had second highest (2.52 ± 0.83).
Khanmohammadi et al., 2015	Between-group, cross-sectional (younger vs. older)	To investigate neurophysiological and biomechanical aspects (including MRCP) of the preparatory postural adjustments during GI in healthy younger and older adults	*n* =31 healthy Younger adults *n* =16 (10F, 26 ±3 years) Older adults *n* =15 (9F, 71 ±3 years)	Cued	Forward step	– MRCP PN occurred earlier (Fz, Cz, Pz) and late MRCP amplitude was smaller (Fz) in older adults vs. younger adults. MRCP PN amplitude did not differ significantly between the two groups
Knaepen et al., 2015	Single-group, cross-sectional, one movement condition	To investigate whether an averaged electrocortical potential could be identified during walking and its temporal relation to the gait cycle.	*n* =10 healthy (7F, 28 ±4 years)	Self-paced	Continuous walking	– Alternating positive and negative potentials occurred twice per gait cycle at Fz and Cz. – Negative peaks corresponded to heel strike. Positive peaks corresponded to push-off.
Peters et al., 2018	Single-group, cross-sectional, multiple movement conditions	To investigate whether motor planning for a voluntary step differs between stepping with the paretic and non-paretic legs, and whether measures of motor planning (the MRCP) are related to EMG and clinical measures of balance and mobility	*n* =13 subacute stroke with lower limb impairment, data included for *n* =10 (4F, 71 ±8 years)	Self-paced	Stepping up onto a 10cm box with either the paretic or non-paretic leg	– Data excluded for *n* =3 post-hoc due to insufficient duration between steps. – No significant differences in MRCP amplitude or duration when stepping the paretic vs. non-paretic legs. – Higher MRCP amplitudes in paretic leg stepping were associated with higher MRCP amplitudes in non-paretic stepping. – For the paretic limb, MRCP amplitude and duration were positively correlated with each other.
Rashid et al., 2018	Single-group, cross-sectional, multiple movement conditions, multiple measurement or processing conditions	To investigate the performance of the ADS1299 EEG device against a high-quality laboratory-based system during both single joint and multi-joint motor tasks	*n* =22 healthy (10F, 36 ±6 years)	Self-paced	Stepping up onto a 23 cm step with the right foot while standing vs. simple ankle DF	– No significant differences between the two EEG systems in signal-to-noise ratio, amplitude and timing of the PN of MRCP, and grand averages of the MRCP. – PN of MRCP was significantly smaller during the stepping task vs. simple ankle DF. Timing of PN did not differ significantly between motor tasks.
Reiser et al., 2020	Single group, cross-sectional, multiple movement conditions	To investigate cognitive-motor interference (utilizing the MRCP) by deploying an auditory cued task-switch paradigm while participants performed a motor task (of increasing complexity).	*n* =23 healthy, included *n* =20 (10F, 19–30 years)	Cued	Performing cognitive task which leads to pressing of left or right response handles while either: standing still, walking in laps, or walking in laps while traversing obstacle course elements.	– MRCP amplitudes for button pressing (in response to cognitive task) were larger while standing vs. walking, but not different for standing vs. obstacle course walking. – Switching cognitive tasks produced larger MRCP amplitudes (and longer response times) for button pressing than repeating the same cognitive task.
Russo et al., 2019	Single-group, cross-sectional, multiple movement conditions, multiple measurement conditions	To investigate whether and how trigger identification techniques (EMG, force plates, and stereophotogrammetry) affect the MRCP in GI	*n* =11 healthy (7F, 22 ±4 years)	Self-paced	Two tasks: Single steps forwards and single steps backwards, alternating left and right feet.	– MRCP amplitude significantly larger for backward stepping than forward stepping. – Significant difference between trigger methods for the mean BP amplitude and amplitude and latency of the peak MRCP. MRCP amplitude was larger when data was time-locked to movement onset using the force plates signal compared to the stereophotogrammetry and EMG.
Sburlea et al., 2015a	Single-group, cross-sectional, one movement condition	To investigate the ability of a BCI to detect the intention to walk in stroke patients from pre-movement EEG correlates (MRCP and ERD) and to investigate how the motivation of patients to execute a task affects the BCI accuracy	*n* =9 chronic stroke (3F, 60 ±11 years)	Self-paced	GI	– Using a detector based on temporal and spectral features (MRCP and ERD), the accuracy for detecting walking intention was 64%. – MRCPs provided higher discrimination between rest and pre-movement states than ERD. – Cz, FC1, and FC2, CP2 were the most discriminative sites for the MRCP. – Higher motivation (according to Intrinsic Motivation Inventory) was correlated with higher detection accuracy.
Sburlea et al., 2015b	Single-group, cross-sectional, one movement condition	To investigate a continuous EEG decoder of a pre-movement state (using MRCP and ERD) in self-initiated walking and the usage of this decoder from session to session without recalibrating	*n* =10 healthy (4F, 26 ±5 years)	Self-paced	GI	– Using a continuous decoder based on a combination of MRCP and ERD features, the accuracy for detecting walking intention was 70%. – Detection accuracy for decoder based on MRCP data was 61%. – For subsequent sessions without recalibration, detection accuracy decreased by 4% after a 1–2 week intersession interval.
Sburlea et al., 2017	Between-group, cross-sectional, one movement condition, multiple measurement or processing conditions	To investigate the instantaneous phase of the MRCP and its application to the detection of GI (by comparing three different detectors of gait intention: MRCP amplitude, MRCP phase, and MRCP amplitude + phase)	*n* =19 Chronic stroke *n* =9 (3F, 60 ±11 years) Healthy *n* =10 (4F, 26 ±5 years)	Self-paced	GI	– Detector based on “MRCP phase” features had the highest accuracy for detecting walking intention (66.5% in healthy and 63.3% in stroke participants.) – For a subsequent session without recalibration, the detector based on “MRCP amplitude + phase” features had the highest detection accuracy (61.0% in healthy and 58.3% in stroke participants), whereas the detection accuracy significantly decreased for the other two detector systems.
Varghese et al., 2016	Single-group, cross-sectional, multiple movement conditions	To investigate the cortical events (MRCP) prior to the mediolateral anticipatory postural adjustment (APA) preceding a lateral stepping task, by comparing the cortical events prior to the focal task of lateral stepping between conditions with and without a preceding APA	*n* =14 healthy (3F, 19–33 years)	Cued	Lateral step starting with equal weight through feet (APA + step condition), lateral step with weight pre-shifted to opposite side (non-APA unloaded step condition), and lateral weight shift only (APA only condition).	– MRCPs response-locked to the APA of “weight shift only” vs. “lateral stepping” were not significantly different. – MRCPs response-locked to foot-off of “lateral step” vs. “pre-shifted lateral step” were significantly different, with a larger MRCP (MP and NS) amplitude for the equal weighted stepping condition.
Vidailhet et al., 1993	Between-group, cross-sectional, multiple movement conditions (simple vs. complex)	To investigate the BP (MRCP) preceding a simple foot movement while sitting and a stepping movement while standing in healthy and PD patients (off medication)	*n* =14 PD *n* =7 (43–55 years) Healthy *n* =7 (41 ±8 years, 23–72 years, gender not reported)	Self-paced	Forward stepping vs. seated ankle DF	– MRCP amplitude was larger in forward stepping vs. seated DF in healthy participants. No significant differences between tasks for PD participants. – MRCP amplitude during stepping and ankle DF was larger in healthy vs. PD participants.
Vidailhet et al., 1995	Between-group, cross-sectional, multiple movement conditions (simple vs. complex)	To investigate the cerebral activity (MRCP) before a voluntary stepping movement in four patients with isolated gait ignition failure	*n* =11 Isolated gait ignition failure *n* =4 (2F, 65–70 years) Healthy *n* =7 (mean 41 years, 23–72 years)	Self-paced	Forward stepping vs. seated ankle DF	– In gait-impaired participants, PN of MRCP occurred earlier in stepping vs. ankle DF. – In healthy participants, MRCP amplitude was larger in stepping vs. ankle DF. – No consistent differences in MRCP amplitudes in gait impaired vs. healthy participants (no statistical analysis).
Yazawa et al., 1997	Single-group, cross-sectional, multiple movement conditions (simple vs. complex)	To investigate the late CNV (MRCP) by employing GI as a response task in the simple reaction time paradigm in healthy participants	*n* =10 healthy males (25 ±6 years)	Cued	GI (at least 3 steps starting with the right foot) vs. seated ankle DF	– Late MRCP amplitude at Cz was significantly larger in GI vs. ankle DF.
**Reach and grasp**						
Boulenger et al., 2008	Single-group, cross-sectional, one movement condition	To investigate the influence of subliminal displays of action verbs, concrete nouns, and strings of consonants, on the concurrent preparation and subsequent execution of a reaching movement (using the MRCP and kinematic parameters).	*n* =25 healthy (mean 28 years), data included for *n* =14. Gender not reported.	Cued	Forward reach from chest to grasp a small object 65 cm away (pinch grip).	– Data excluded for *n* =11 due to learning effects, no MRCP, or noisy signals. – MRCP amplitude was significantly smaller in the action verb condition vs. the concrete noun condition.
De Oliveira et al., 2012	Single-group, cross-sectional, one movement condition	To investigate the readiness potential (MRCP) preceding the interaction with emotionally laden stimuli.	*n* =17 healthy males (28 ±4 years), data included for *n* =11.	Self-paced	Forward reach to grasp a transparent cylinder in tray socket (containing pleasant, neutral, and unpleasant items).	– Data excluded for *n* =6 due to >50% of epochs with amplitude exceeding ± 100 μV, no MRCP, or MRCP amplitude >3SDs of group average. – MRCP amplitude was significantly larger for unpleasant stimuli vs. neutral or pleasant stimuli. – MRCP amplitude was significant smaller for pleasant stimuli vs. neutral stimuli.
Eilbeigi and Setarehdan, 2018a	Single-group, cross-sectional, multiple movement conditions, multiple measurement or processing conditions	To (i) investigate the existence of neural correlates of intention to replace an object on the table during a holding phase, and (ii) present a new method, Global optimal constrained ICA (GocICA) to extract the MRCP from a single-trial EEG signal.	*n* =12 healthy (8F, 19–35 years)	Cued	Two components: (i) Forward reach to grasp object with pincer grip and lift it to a specified height, and then (ii) hold for minimum 2 s and then on signal replace object back on table.	– MRCP onset was ≈2 s prior to movement onset for initial reach, and ≈1 s prior to movement for replacing the object. – Using pseudo-online online classification, the accuracy for detecting movement intention was significantly higher with the Charged System Search GocICA method compared to other methods, for both reaching (TPR 92 ±7%) and replacing (TPR 90 ±6%) the object. FPRs were also lowest with this method.
Eilbeigi and Setarehdan, 2018b	See Eilbeigi and Setarehdan, 2018a	To investigate the GocICA algorithm (applied to MRCP analysis) for overcoming the limitations of conventional cICA.	See Eilbeigi and Setarehdan, 2018a	Cued	See Eilbeigi and Setarehdan, 2018a	– For offline single trial MRCP analysis, the accuracy for detecting intention to reach was significantly higher with the GocICA method, compared to cICA and two ICA-based methods. The highest accuracy was obtained with the Charged System Search GocICA method (TPR of 91 ±3% and FPR of 9 ±4%).
Koester and Schack, 2016	Single-group, cross-sectional, multiple movement conditions	To investigate whether specific motor representations for grip types interact neurophysiologically with conceptual information (by examining movement parameters and ERPs, including the MRCP).	*n* =28 native German speakers, data included for *n* =26 (15F, 20–30 years)	Cued	In response to word presentation, reach, grasp and lift the object in front of the word using either a precision or power grip (hold for 1–2 s), then replace object. Words denoted objects requiring different grips and could be (in)congruent with the task.	– Data for *n* =2 excluded due to movement artifacts. – MRCP amplitude was larger when presented with words denoting large objects vs. words denoting small objects, and when performing precision grip vs. power grip.
Kourtis et al., 2013	Single-group, cross-sectional, multiple movement conditions	To investigate whether an individual represents and simulates the action of an interacting partner when planning to perform a joint action (by examining ERPs, including MRCP)	*n* =16 healthy (8 pairs, 9F, 26 ±7 years)	Cued	Partner A reaching and lifting an object and replacing it, vs. passing it to partner B who replaces the object.	– Timing of PN of MRCP of partner B corresponded more closely to the onset of partner A’s action than to the onset of Partner B’s action.
Schwarz et al., 2018	Single-group, cross-sectional, multiple movement conditions	To investigate three different executed reach-and-grasp actions (palmar, pincer and lateral grasps) utilizing their EEG neural correlates (including the MRCP).	*n* =15 healthy (8F, 23–37 years)	Cued	Reach, grasp, pick up, and hold an object while the tile underneath was illuminated and then replace it. Objects were a glass (palmer grasp), a needle (pincer grasp) and a key in a keyhole (lateral grasp).	– Strong negative shift 250–350 ms prior to movement onset that peaked near movement onset, and a second smaller negative peak ≈400 ms after movement onset. – Timing of the positive peak of the second positive rebound was significantly earlier for the lateral grasp vs. pincer or palmar grasp conditions. – A classification accuracy of 65.9 ±8.1% was obtained for a 4-class classification problem (no movement vs. pincer grasp vs. palmar grasp vs. lateral grasp).
Schwarz et al., 2020a	Between-group, cross-sectional, multiple movement conditions, multiple measurement or processing conditions	To investigate whether EEG-based correlates of natural reach-and-grasp actions can be successfully identified and decoded using two mobile EEG systems (compared with gold standard).	3 groups of *n* =15 healthy (15–30 years): gel-based gold standard (5F), water-based electrode system (8F), dry electrode system (7F).	Self-paced	Reach to grasp of two different objects with right hand (palmar grasp of empty jar or lateral grasp of spoon)	– No significant differences in MRCP morphology between palmar grasp vs. lateral grasp. – Gel- and water-based electrode systems had similar MRCP morphology (as per Schwarz et al., 2018), however dry electrode system had smaller MRCP amplitude with attenuated peaks. – Offline single trial classification (3 classes) was lower for dry electrodes. However, when all EEG systems were reduced to 11 electrodes, there was no difference between systems.
Schwarz et al., 2020b	Single-group, cross-sectional, multiple movement conditions	To investigate the neural correlates of unimanual and bimanual reach-and-grasp actions using low-frequency time-domain EEG (MRCPs).	*n* =15 healthy (21–30 years, gender not reported)	Self-paced	Reach to grasp either unimanually with left or right hand (palmar grasp of a jar and lateral grasp of a spoon) or bimanually (double lateral grasp of pot handles and mixed grasping of jar and spoon).	– MRCP shape as per Schwarz et al. (2018) with initial negative shift ≈500 ms prior to movement onset and second smaller negative peak ≈250 ms after movement onset. – Topography of MRCPs was significantly different between unimanual vs. bimanual, and left vs. right-handed tasks; likely due to lateralization effects.
						– PN amplitude not significantly different between unimanual vs. bimanual conditions. – Accuracy for detecting movement type from all 6 movement classes was 30–41%.
Zaepffel and Brochier, 2012	Single-group, cross-sectional, multiple movement conditions	To investigate the planning processes (including the MRCP) of reach-to-grasp movements using a pre-cuing task (where different instructions were given for grasp type and force level).	*n* =14 healthy (9F, 21–41 years)	Cued	Reach, grasp and pull of a knob situated in front of the participant, using either a pincer or key grip, and either high or low force.	– Providing pre-cuing information about force or grip increased the late MRCP amplitude vs. providing no information. – MRCP amplitude varied according to the different pre-cuing instructions and across different scalp locations.
**Virtual Driving**						
Bayliss and Ballard, 2000	Single-group, cross-sectional, multiple movement conditions	To investigate on-line recognition (of the P3 and MRCP) in the virtual reality environment (virtual driving).	*n* =5 healthy (19–52 years)	Cued	Pressing of brake pedal on a go-kart which controlled a virtual car, in response to changing yellow, red, and green lights.	– MRCP onset observed ≈2 s before the light changed from yellow to red or green, indicating slowing down and preparation for breaking.
Khaliliardali et al., 2012	Single-group, cross-sectional, multiple movement conditions, multiple measurement or processing conditions	To investigate anticipatory brain signals (MRCPs) and evaluate the discriminability of these potentials using single trial classification methods (during driving simulation).	*n* =6 healthy (1F, 24–32 years)	Cued	Pressing the gas or brake pedal to “Go” or Stop” while driving a car simulator with a virtual roadway.	– Observed MRCP onset ≈1 s before Go/Stop cue. PN aligned with Go/Stop cue and had larger amplitude for Stop trials. – Offline analysis showed slightly better specificity for QDA compared to linear discriminant analysis (77 ±11% for Go and 78 ±5% for Stop trials), and vice versa for sensitivity (62 ±13% for Go and 73 ±13% for Stop trials).
Khaliliardali et al., 2015	Single-group, cross-sectional, multiple movement conditions, multiple measurement or processing conditions	To investigate the neural signatures (MRCPs) of anticipation of specific actions, namely braking and accelerating.	*n* =18 healthy (2F, 26 ±4 years)	Cued	See Khaliliardali et al., 2012	– Offline single trial classification using QDA classifiers had TPR of 79 ±12% for Go and 83 ±13% for Stop trials – Classification with single vs. multiple electrodes had similar performance.
Moinnereau et al., 2019	Single-group, cross-sectional, one movement condition, multiple measurement or processing conditions	To investigate three EEG artifact removal algorithms tailored for MRCP detection while driving, and propose two machine learning methods, recurrent neural network (RNN) reservoir and a support vector machine (SVM), for predicting intent to change lanes.	*n* =5 healthy (age/gender not reported)	Self-paced	Performing a series of left and right lane change maneuvers in a car (i.e., use of a steering wheel).	– For single trial performance EMG and accelerometer-based ICA artifact removal outperformed constrained ICA. – Accuracy for detecting intention to change lanes to left or right was highest with the RNN method (mean accuracy within 2 s before lane changing of 83%) vs. SVMs (54%). – Increased processing window length improved recognition rates; a window >3 s provided sufficiently reliable classification.
Welke et al., 2011	Single-group, cross-sectional, multiple movement conditions	To investigate the MRCP to identify the onset of the anticipated activation within motor areas of the brain due to steering maneuvers.	*n* =14 healthy (3F, 26 ±3 years)	Cued	Left and right turns in a car (i.e., use of a steering wheel).	– MRCP onset observed ≈190 ms prior to steering action onset and was not significantly different between left and right turns.
**Sit to Stand**						
Bulea et al., 2014	Single-group, cross-sectional, multiple movement conditions	To investigate the ability to decode movement intention from delta-band EEG (MRCP) recorded immediately before movement execution in healthy volunteers (using sit-to-stand and stand-to-sit movements, and self-initiated and cued paradigms).	*n* =10 healthy (4F, aged not reported)	Self-paced and cued	Sit-to-stand and stand-to-sit from chair	– In 3/10 participants, MRCPs were more prominent in self-paced vs. cued movement. – Using an LFDA-GMM classifier, accurate classification into sit-to-stand, stand-to-sit and quiet periods was 71.8%, 66.7%, and 83.7% in cued scenario, and 75.8%, 70.6%, and 87.5% in self-paced scenario. There was no significant difference between cued and self-paced.
Chaisaen et al., 2020	Single-group, cross-sectional, multiple movement conditions	To investigate the decoding of continuous EEG rhythms during action observation, motor imagery, and motor execution for the actions of standing and sitting.	*n* =8 healthy (5F, 20–29 years)	Cued	Sit-to-stand, stand-to-sit. Imagined and executed.	– MRCP PN occurred earlier for sit-to-stand vs. stand-to-sit. – TPR for movement execution vs. rest was 65.7 ±2.7% for sit-to-stand and 72.3 ±2.5% for stand-to-sit. For imagined movement it was 65.4 ±3.9% for sit-to-stand and 70.9 ±4.4% for stand-to-sit. – FPR was significantly higher for executed than imagined movements (42.7 ±1.7% vs. 15.5 ±1.6% for sit-to-stand and 51.4 ±5.0% vs. 16.3 ±1.8% for stand-to-sit).
Jacobs et al., 2011	Single-group, cross-sectional, multiple movement conditions	To investigate changes in postural coordination and pre-movement cerebrocortical activity (MRCP) related to the experience of acutely-induced low back pain (LBP).	*n* =14 healthy with no history of LBP (8F, mean 28 years, 19–48 years)	Cued	Sit-to-stand with three ordered conditions: (i) no pain, (ii) electrically-induced LBP, and iii) no pain after the painful condition.	– No main effect of condition on MRCP amplitude; however post-hoc test showed increased MRCP amplitude at C4 in LBP condition vs. no pain condition. – Altered movement parameters in the LBP condition significantly correlated with increased MRCP amplitude at C4.
Jochumsen and Niazi, 2020b	Described in “Walking related” section					
Singh et al., 2016	Single-group, cross-sectional, one movement condition, multiple measurement or processing conditions	To investigate the MRCP related to the rise of stand-up from the seated position	*n* =8 healthy males (27 ±3 years)	Cued	Sit-to-stand	– When time-locked to the gyro sensor, PN of MRCP occurred 1305 ms later than when time-locked to quadriceps EMG; this was comparable with the time difference between the onsets of the gyro sensor and EMG (1157 ms).

*APA, anticipatory postural adjustment; BP, Bereitschaftspotential; DF, dorsiflexion; ERD, event-related desynchronization; F, female; FPR, false positive rate; GI, gait initiation; GocICA, global optimal constrained ICA; ICA, independent component analysis; LL, lower limb; M, male; MRCP, movement-related cortical potential; PD, Parkinson’s disease; PN, peak negativity; QDA, quadratic discriminant analysis; RP, readiness potential; RBSE, reference based source extraction; TPR, true positive rate; UL, upper limb; vs., versus. # Note MRCP was filtered out with high-pass filter (5 Hz).*

**TABLE 4:** Experimental studies.

Author	Study design	Aim of study	Participants	Mode	Movement task	Purpose of MRCP	Key findings
Barthel et al., 2001	Randomized cross-over trial	To investigate the influence of caffeine/taurine and physical stress on the cortical movement preparation preceding voluntary self-paced pedaling.	*n* = 15 male endurance cyclists (26 ± 3y), data included for *n* = 14	Self-paced	Right leg pedaling movement on cycle ergometer	Outcome measure following single intervention session	– Data excluded for *n* = 1 due to artifacts – With increased physical exertion, MRCP amplitude increased. – Distribution and magnitude of MRCP changes differed for ‘caffeine’ and ‘caffeine + taurine’ conditions.
Fromer et al., 2016	Non-randomized controlled trial	To investigate the effect of training schedule (blocked or random) on learning-related changes in preparatory brain activity	*n* = 120 healthy (60F, 25 ± 6y), divided into 2 equally skilled groups to complete blocked or randomized training.	Cued	Dart throwing using Wii remote	Outcome measure following single training session	– MRCP amplitude decreased with increasing performance. – During training, MRCP amplitude was significantly larger for random training vs. blocked training.
Mizusaki et al., 2019	Randomized controlled trial	To investigate whether Quiet Eye training is associated with motor preparation processes by using MRCPs.	*n* = 18 male students (22 ± 2y), data included for *n* = 12, randomized to Quiet Eye training or Control training.	Self-paced	Seated dart throwing at a dartboard	Outcome measure following nine training sessions over 3 weeks.	– Data excluded for *n* = 6 due to insufficient EEG data. – Both Quiet Eye training and Control training had improved performance, but there were no differences in MRCP amplitudes between training groups.
Mrachacz-Kersting et al., 2019a	Non-randomized cross-over trial	To investigate the excitability of the cortical projections to an upper extremity muscle in healthy participants following a single session of the associative BCI (using simple vs. complex movements).	*n* = 7 healthy (5F, 21–32y)	Cued	Reach-to-grasp (exact parameters not stated) vs. wrist extension	Component of BCI intervention (to time electrical stimulus)	– Observed larger increases in corticomotor excitability to the extensor carpi radialis muscle following the BCI intervention using simple movement vs. complex reaching movement; however, there was no statistical analysis.
Peters et al., 2020	Subset from randomized controlled trial	To investigate whether motor planning deficits can be altered via fast stepping retraining or conventional physical therapy in individuals in the subacute stage after stroke.	*n* = 7 subacute stroke, randomized to fast stepping (*n* = 4, 2F, 63–69y) or conventional physical therapy (*n* = 3, 0F, 73–84y)	Self-paced	Stepping onto 10 cm high box with either paretic or non-paretic leg	Outcome measure following 12 intervention sessions	– Observed decrease in MRCP duration for paretic and non-paretic stepping following both interventions; however, there was no statistical analysis. – MRCP amplitude changes were variable.
Wright et al., 2012a	Non-controlled trial	To investigate the effect of ecologically valid motor skill training (guitar playing) on cortical activity related to motor planning	*n* = 10 non-musicians (5F, 26 ± 9y)	Self-paced	Guitar playing (G major scale)	Outcome measure following 5-week training program	– MRCP amplitude at C3 and CZ was significantly smaller post training.

*BCI, brain computer interface; F, female; M, male.*

#### Study Designs

Within the 59 studies included, 53 studies used an observational design, while six used an experimental paradigm. Of the observational research, 25 studies utilized a single group, cross-sectional, multiple condition protocol, where two or more movements were compared in the same participants (Jung, 1982; Yazawa et al., 1997; Bayliss and Ballard, 2000; Do Nascimento et al., 2005; Jacobs et al., 2011; Welke et al., 2011; Fromer et al., 2012; Zaepffel and Brochier, 2012; Kourtis et al., 2013; Bulea et al., 2014; Jiang et al., 2015; Euler et al., 2016; Koester and Schack, 2016; Varghese et al., 2016; Martinez-Exposito et al., 2017; Peters et al., 2018; Schwarz et al., 2018; Nann et al., 2019; Berchicci et al., 2020; Braquet et al., 2020; Chaisaen et al., 2020; Reiser et al., 2020; Tomyta and Seki, 2020; Jochumsen and Niazi, 2020b; Schwarz et al., 2020b). Six studies utilized a single group, cross-sectional, single condition protocol, where only one movement task was investigated (Boulenger et al., 2008; De Oliveira et al., 2012; Knaepen et al., 2015; Sburlea et al., 2015a,b; Bodda et al., 2020). Ten studies utilized a between-group, cross-sectional design, where two groups (e.g., experts versus novices) performed one movement task (Mann et al., 2011; Wright et al., 2012b; Khanmohammadi et al., 2015; Vogt et al., 2017; Skrzeba and Vogt, 2018), or where two groups performed multiple movement tasks (O’connor, 1986; Vidailhet et al., 1993, 1995; Berchicci et al., 2017; Fearon et al., 2021). Twelve studies investigated multiple EEG measurement or signal processing techniques (Khaliliardali et al., 2012, 2015; Singh et al., 2016; Jeong et al., 2017; Sburlea et al., 2017; Rashid et al., 2018; Eilbeigi and Setarehdan, 2018a,b; Karimi and Jiang, 2019; Moinnereau et al., 2019; Russo et al., 2019; Schwarz et al., 2020a). The six experimental studies consisted of two randomized controlled trials (Mizusaki et al., 2019; Peters et al., 2020), one non-randomized controlled trial (Fromer et al., 2016), one randomized cross-over trial (Barthel et al., 2001), one non-randomized cross-over trial (Mrachacz-Kersting et al., 2019a), and one non-controlled trial (Wright et al., 2012a).

#### Participants

Within the 59 included studies, 50 investigated healthy populations, one investigated healthy young and older adults (Khanmohammadi et al., 2015), and four investigated both healthy and clinical populations [stroke (Sburlea et al., 2017), Parkinson’s Disease (PD) (Vidailhet et al., 1993; Fearon et al., 2021), and gait-ignition failure syndrome (Vidailhet et al., 1995)]. A further three studies investigated stroke only populations (Sburlea et al., 2015a; Peters et al., 2018; Peters et al., 2020) and one study investigated smokers (O’connor, 1986). Sample sizes ranged from 2 to 120 participants and ages ranged from 18 to 84 years.

#### Movement Tasks Generating the Movement-Related Cortical Potential

There were a wide range of movement tasks investigated. These movements could be categorized into five groups: (1) specialized goal-directed activities, which included movement tasks such as juggling, guitar playing, golf putting, and bungee jumping, (2) walking-related tasks, (3) reach and grasp, (4) virtual driving, and (5) sit-to-stand.

Many of the articles categorized under ‘walking-related’ or ‘sit-to-stand’ activities had an overarching aim focused on developing BCI-assistive robotic devices to aid their respective task (Do Nascimento et al., 2005; Bulea et al., 2014; Jiang et al., 2015; Sburlea et al., 2015a,b, 2017; Singh et al., 2016; Jeong et al., 2017; Karimi and Jiang, 2019; Chaisaen et al., 2020; Jochumsen and Niazi, 2020b). While the virtual driving literature often focused on the development of intelligent cars (Welke et al., 2011; Khaliliardali et al., 2012, 2015; Moinnereau et al., 2019). The reach-to-grasp literature primarily aimed to investigate MRCP differences between various reach/grasp types or task goals (De Oliveira et al., 2012; Koester and Schack, 2016; Schwarz et al., 2018, 2020a,b). This was mainly to investigate if the MRCP could be used to differentiate between similar movement tasks. Additionally, articles using reach-to-grasp movements investigated the MRCP under different environmental conditions (Boulenger et al., 2008; Zaepffel and Brochier, 2012; Kourtis et al., 2013; Koester and Schack, 2016), such as when reaching for emotionally unpleasant objects (De Oliveira et al., 2012).

#### Mode (Self-Paced Versus Cued)

A similar number of studies used self-paced movement (*n* = 28) compared to cued movement (*n* = 28), with three studies investigating both (Jung, 1982; Bulea et al., 2014; Tomyta and Seki, 2020). Two studies compared self-paced with cued movements and found no significant differences in MRCP characteristics (Bulea et al., 2014; Tomyta and Seki, 2020).

### Observational Research: Movement-Related Cortical Potential Characteristics

#### Movement Complexity and Expertise

Of the observational research, eight studies compared differences between simple and complex movements (e.g., ankle dorsiflexion compared with forward stepping, 1-ball versus 2-ball juggling, large-target versus small-target throwing) (Vidailhet et al., 1993, 1995; Yazawa et al., 1997; Fromer et al., 2012; Berchicci et al., 2017; Martinez-Exposito et al., 2017; Rashid et al., 2018; Tomyta and Seki, 2020). In six of these eight studies, MRCP amplitudes were significantly larger in more complex movement tasks (Vidailhet et al., 1993, 1995; Yazawa et al., 1997; Fromer et al., 2012; Berchicci et al., 2017; Tomyta and Seki, 2020). One study found the prefrontal MRCP onset was earlier in more complex juggling movements in both expert jugglers and novices (Berchicci et al., 2017). In contrast, a study that compared step-ups with simple ankle dorsiflexion in healthy participants found a larger peak negativity for the simple movement, and no difference in the peak negativity timing (Rashid et al., 2018).

Movement complexity was also manipulated with the use of targets. For example, Fromer et al. found that simulated dart throwing with a Wii remote resulted in larger MRCP amplitudes when aiming for a small target (increased difficulty) compared with a large target (Fromer et al., 2012). Jung compared slow finger pointing and rapid punching to the same target and observed a longer MRCP duration for the slow pointing condition (Jung, 1982).

The interaction between movement task complexity and an individual’s stage of learning was investigated in five studies which compared novices and experts performing tasks such as juggling (Berchicci et al., 2017), golf putting (Mann et al., 2011), badminton serving (Skrzeba and Vogt, 2018), archery (Vogt et al., 2017), and guitar playing (Wright et al., 2012b). Four of five studies found larger MRCP amplitudes in the expert groups (Mann et al., 2011; Berchicci et al., 2017; Vogt et al., 2017; Skrzeba and Vogt, 2018), while one study found smaller MRCP amplitudes in experts (Wright et al., 2012b). Two of these studies also investigated MRCP latency and showed a later MRCP onset in skilled versus non-skilled archers (Vogt et al., 2017) and a later MRCP negative slope in expert guitar players compared to novices (Wright et al., 2012b).

#### Reach and Grasp

Several studies investigated MRCPs during different grasp types. Unimanual and bimanual reach-to-grasp movements showed the characteristic peak negativity near movement onset, but also had a second smaller negative peak approximately 250–400 ms after movement onset (Schwarz et al., 2018, 2020a,b). Schwarz et al. found no differences in MRCP amplitudes between unimanual versus bimanual tasks or between lateral grasps (of a spoon) and palmar grasps (of a jar) (Schwarz et al., 2020a,b), whereas Koester and Schack found larger MRCP negativity 100–300 ms after movement onset with a two-finger precision grasp (of a small cube) versus a palmar grasp (of a large cube) (Koester and Schack, 2016). Schwarz et al. found differences in MRCP timing with different grasp types; the positive rebound after the second negative peak (which coincided with the completion of the grasp movement) occurred earlier for key grasps compared with pincer or palmer grasps (Schwarz et al., 2018).

#### Walking and Mobility

Several studies compared the MRCP under different stepping conditions. Gait initiation or stepping in the backward direction produced larger amplitude MRCPs than the forward direction (Do Nascimento et al., 2005; Russo et al., 2019; Berchicci et al., 2020). Forward gait initiation produced smaller amplitude MRCPs than step-up, side-step, backward step, and stand-to-sit movements (Jochumsen and Niazi, 2020b). In addition, lateral stepping produced smaller MRCPs if the weight was pre-shifted to the supporting leg than if the weight was equally distributed prior to stepping (Varghese et al., 2016).

#### Driving

Studies that investigated MRCPs during virtual driving could identify an MRCP prior to the presentation of Go and Stop signals, a peak negativity aligning with the Go/Stop signals, and a larger peak negativity for Stop versus Go movements (Bayliss and Ballard, 2000; Khaliliardali et al., 2012). MRCP onsets occurred approximately 190ms prior to left and right turns, with no differences between the two (Welke et al., 2011).

#### Attention and Emotion

Several studies investigated the effects of different attentional loads by manipulating the visual cue. Disorientating visual cues during a stepping task did not alter MRCP amplitude or latency (Braquet et al., 2020), but unfamiliar complex visual cues during an upper limb movement sequence produced a smaller MRCP amplitude and later peak negativity (Euler et al., 2016). Subliminal exposure to action verbs versus concrete nouns during movement preparation of a reach-to-grasp movement produced a smaller MRCP amplitude (Boulenger et al., 2008). Whereas, providing a visual cue denoting the force level and grasp type required (3 s prior to the Go signal), produced a larger MRCP amplitude than providing no cuing information (Zaepffel and Brochier, 2012).

Two studies manipulated attention under a dual-tasking paradigm, where the MRCP was recorded during a simple hand movement while participants also performed a walking task (Reiser et al., 2020; Fearon et al., 2021). In one study, participants performed a cognitive task that resulted in pressing a response handle while also performing a secondary walking task; the MRCP amplitudes for the hand movement were smaller while walking compared to standing still (Reiser et al., 2020). In the second study, healthy participants or people with PD performed a button pressing task while either sitting or walking in place; the MRCP duration was longer during the dual-task condition in people with PD and freezing of gait, compared to people with PD without freezing of gait and healthy controls (Fearon et al., 2021).

Attention was also manipulated by performing a shared task with a partner; during a shared task where one person picked up and passed an object to another person, the peak negativity of the MRCP of the person receiving the object aligned more closely with the movement onset of the person who had picked up the object than their own movement onset (Kourtis et al., 2013). In terms of emotional loads, reaching for emotionally unpleasant stimuli produced larger amplitude MRCPs (De Oliveira et al., 2012), but a 192-m bungee jump produced the same MRCP onset and amplitude as jumping off a 1-m platform in two professional cliff divers (Nann et al., 2019).

#### Aging and Neurological Conditions

When comparing younger and older adults performing a forward step, older adults had an earlier MRCP peak negativity and a smaller amplitude of the late MRCP (Khanmohammadi et al., 2015). When comparing healthy individuals with people with PD, MRCP amplitudes were smaller in the PD group during both gait initiation and seated dorsiflexion (Vidailhet et al., 1993). However, the MRCP for a simple hand movement when recorded under a walking dual-task condition, had a larger amplitude in people with PD and freezing of gait, compared to people with PD without freezing of gait and healthy controls (Fearon et al., 2021). When comparing healthy individuals with those with isolated gait ignition failure, no consistent differences in MRCP amplitudes were observed; although this was a small descriptive study (Vidailhet et al., 1995). Of the four studies that investigated stroke populations, none specifically compared the MRCP characteristics between healthy and stroke participants. However, one study compared step-ups with the more-affected and less-affected legs and found no differences in MRCP amplitude or duration (Peters et al., 2018).

### Movement-Related Cortical Potential Extraction, Detection, and Classification

Ten articles investigated different EEG measurement or processing systems during mobility-related tasks (Bulea et al., 2014; Sburlea et al., 2015a,b, 2017; Jeong et al., 2017; Rashid et al., 2018; Karimi and Jiang, 2019; Russo et al., 2019; Chaisaen et al., 2020; Jochumsen and Niazi, 2020b). Findings from all but one of these studies (Russo et al., 2019) demonstrated that the MRCP signal could be successfully classified or enhanced using multiple techniques (this was not the aim of Russo et al., 2019). Accuracy rates varied when using the different signal processing techniques, but similar accuracies were reported when comparing healthy and stroke participants (Sburlea et al., 2015b, 2017). Five articles in the reach-to-grasp dataset examined different EEG measurement or processing techniques (Eilbeigi and Setarehdan, 2018a,b; Schwarz et al., 2018, 2020a,b). Schwarz et al. found a dry electrode system produced a lower amplitude MRCP with attenuated peaks compared to gel- or water-based electrodes (Schwarz et al., 2020a). Eilbeigi and Setarehdan found global optimized constraint independent component analysis (GocICA) more effective at denoising the EEG for enhancing multichannel EEG signal detection of the MRCP compared to constrained independent component analysis (cICA) (Eilbeigi and Setarehdan, 2018a,b). Schwarz et al. found binary single-trial classification to be superior to multiclass single-trial classification, with accuracy rates of 93.5 and 65.9%, respectively (Schwarz et al., 2018). Similarly, the virtual driving research focused on analysis of EEG processing algorithms used to remove excess signal noise (Khaliliardali et al., 2012, 2015; Moinnereau et al., 2019). For example, Moinnereau et al. (2019) compared accelerometer-based ICA, cICA and empirical model decomposition (EMP) analysis, and found that denoising with cICA led to the highest classification accuracy. They also found a processing window of greater than 3 s sufficient to provide reliable classification. Two studies (Singh et al., 2016; Russo et al., 2019) compared the effect of different means of synchronization. It was reported that the peak negativity of the MRCP occurred earlier when synchronizing the EEG to EMG compared with gyroscope data (Singh et al., 2016), while synchronization of the EEG using a force plate was associated with MRCPs of greater amplitude compared to synchronization with EMG or stereophotogrammetry (Russo et al., 2019).

### Experimental Research

Six studies had an experimental design (Table 4), with one study using the MRCP as part of their intervention (Mrachacz-Kersting et al., 2019a). This associative BCI-intervention was applied to seven healthy participants and involved timing radial nerve electrical stimulation to the MRCP generated during either a simple wrist extension task or a complex reach-to-grasp task. Increased corticomotor excitability was observed with the simple wrist extension condition, although no statistical analysis was performed (Mrachacz-Kersting et al., 2019a).

The remaining five experimental studies used the MRCP as an outcome measure to determine the effect of an intervention. The movement tasks used to measure the MRCP were all used as part of the interventions, for example; pedaling (Barthel et al., 2001), dart throwing (Fromer et al., 2016; Mizusaki et al., 2019), guitar playing (Wright et al., 2012a), and step-ups (Peters et al., 2020). Two studies showed a decrease in MRCP amplitude with improved performance in dart throwing (Fromer et al., 2016) and guitar playing (Wright et al., 2012a). Another study showed a decrease in MRCP amplitude with physical exertion over a single session (Barthel et al., 2001). The only experimental study that was undertaken with participants with stroke (*n* = 7) observed a decrease in the duration of MRCPs recorded during step-ups following 12 training sessions (physical therapy or fast stepping training), although there was no statistical analysis (Peters et al., 2020). A final study investigated MRCP changes following nine dart throwing sessions using eye gaze training or control training; they reported no significant between-group differences in MRCP amplitude, but did not report within-group changes (Mizusaki et al., 2019).

## Discussion

This review is the first of its kind in this field and offers researchers and clinicians important insight into the breadth of research investigating the MRCP during ecologically valid movements. The following discussion focuses on: the characteristics of the MRCP in various populations and under various task conditions, the potential use of the MRCP as an outcome measure, and the application of the MRCP within rehabilitation interventions.

### Observational Research: Movement-Related Cortical Potential Characteristics

This review identified a number of studies that compared the MRCP during complex and simple movements. In general, larger MRCP amplitudes were seen with more complex movements, for example in 2-ball versus 1-ball juggling (Berchicci et al., 2017), dart throwing movement versus button release (Fromer et al., 2012), 4-finger tapping versus 1-finger tapping or drum-stick tapping (Tomyta and Seki, 2020), forward stepping versus ankle dorsiflexion (Vidailhet et al., 1993, 1995), and gait initiation versus ankle dorsiflexion (Yazawa et al., 1997). This aligns with the understanding that MRCPs reflect motor planning processes, and that more difficult tasks elicit cortical responses of greater magnitude, thereby generating larger MRCP amplitudes (Shibasaki and Hallett, 2006). This is further supported by studies of walking tasks that found that more complex gait tasks, such as backward walking, backward stepping, and forward step-ups, produced larger MRCP amplitudes than forward stepping or walking (Do Nascimento et al., 2005; Russo et al., 2019; Berchicci et al., 2020; Jochumsen and Niazi, 2020b). In contrast, one study showed smaller MRCP amplitudes during step-ups compared with simple ankle dorsiflexion (Rashid et al., 2018). The reason for these contrasting findings is not clear and does not appear to be related to differences in study design, but readers should consider that this body of evidence is small and that factors such as motivation, effort, and the kinematics of the movement may influence the findings (Do Nascimento et al., 2006; Shibasaki and Hallett, 2006; Jochumsen et al., 2013).

Previous research on simple movements has established the idea that as an individual develops expertise in a movement task, the relative difficulty of that task decreases and the associated MRCP amplitude is smaller (Wright et al., 2011). This review of more complex movements included one study that reflected this; during a guitar-playing task, experienced guitarists had smaller-amplitude MRCPs than non-musicians (Wright et al., 2012b). However, we also identified four studies in which experts demonstrated larger-amplitude MRCPs than novices during tasks such as juggling (Berchicci et al., 2017), golf putting (Mann et al., 2011), badminton serving (Skrzeba and Vogt, 2018), and archery (Vogt et al., 2017). These contrasting findings may be explained by the different task requirements. The smaller-amplitude MRCP was observed when expert guitarists played the G-major scale which may reflect the relative automaticity of this task in these experts, where they likely required less cognitive workload than non-musicians to manipulate the guitar strings in a seated position (Wright et al., 2012b). Whereas, the motor tasks that produced larger MRCPs in experts (juggling, golf, badminton, archery) have a high degree of uncertainty and require a high level of motor control and precision; thus, the increased performance of experts in these more complex tasks appeared to be associated with greater activation of motor preparation areas, due to the multisensory integration required. The increased motor preparation required for target-based tasks such as badminton and juggling aligns with findings of Fromer et al. where simulated dart throwing with a small target (i.e., a more difficult task) produced larger amplitude MRCPs than a large target (Fromer et al., 2012).

A number of studies manipulated various factors related to attention in order to understand the MRCP under different task conditions. The presentation of an unfamiliar complex visual cue prior to an upper limb sequence task produced a smaller MRCP amplitude and later peak negativity (Euler et al., 2016). Similar findings have been observed during simple movements. For example, smaller MRCP amplitudes have been recorded when a cognitively demanding task preceded a button-pushing movement (Baker et al., 2011) and when an attention-diverting auditory task was performed concurrently with an ankle dorsiflexion task (Aliakbaryhosseinabadi et al., 2017). This might suggest that increasing the cognitive load associated with a movement task reduces resources available for motor preparation (Baker et al., 2011). However, other research findings in this review contrasted with this. Disorientating visual cues during a stepping task did not affect the MRCP (Braquet et al., 2020), perhaps because of the automatic nature of the stepping task (Clark, 2015), and the provision of additional cues about the type of upcoming grasp movement produced a larger MRCP amplitude (Zaepffel and Brochier, 2012). These combined results might suggest that when the cognitive conditions of the task increase attention onto the upcoming complex motor task there is an increase in motor preparation, whereas when the cognitive load diverts attention away from the movement task there is a reduction in motor preparation. The resources available for motor preparation can also be reduced by adding a secondary physical task as seen when the MRCP recorded during a simple hand movement had a smaller amplitude when participants performed a concurrent walking task compared to standing still (Reiser et al., 2020). When a similar dual-tasking paradigm was performed by people with PD and healthy controls, there was no difference in MRCP amplitude between single and dual-task conditions, but people with PD and freezing of gait had a longer MRCP duration under the dual-task condition (Fearon et al., 2021), suggesting that the addition of the secondary walking task altered the cortical processes required for the preparation of a simple hand movement. These findings related to the presence of concurrent cognitive or physical task demands may have implications for neurological rehabilitation where patients frequently experience impairments in attention (Rabinowitz and Levin, 2014; Loetscher et al., 2019) and where the rehabilitation environment can be noisy and distracting. Therapists should consider whether the presence of attention-diverting stimuli is hindering the patients ability to activate motor cortical areas, or in contrast, whether it presents a useful challenge to their motor planning when incorporated into a dual-task training program (Fritz et al., 2015).

In terms of emotional stimuli, MRCP amplitudes were larger when reaching for unpleasant compared with pleasant objects (De Oliveira et al., 2012), reinforcing that the MRCP is susceptible to task-related factors. Given this effect, it was surprising that two cliff divers produced comparable MRCPs when jumping off a 1-m platform and bungee jumping from 192 m (Nann et al., 2019), however, their familiarity with this task may have reduced their perception of risk and limited the influence of their emotions. Previous literature has established that MRCP characteristics during simple movement tasks are modulated by emotional and stressful stimuli, and also by the individuals level of anxiety (Knott and Irwin, 1973; Glanzmann and Froehlich, 1984; Perri et al., 2014). This area requires further research in ecologically valid movements to enable clinicians to understand whether emotionally stimulating environments facilitate or inhibit motor preparation and execution.

An important finding of this review is the scarcity of studies investigating MRCPs in people with clinical conditions. The seven observational studies in clinical or older adult populations investigated forward stepping, gait initiation, step-ups, or used walking as a secondary task (Vidailhet et al., 1993, 1995; Khanmohammadi et al., 2015; Sburlea et al., 2015a, 2017; Peters et al., 2018; Fearon et al., 2021), which is encouraging given the strong focus on mobility tasks in rehabilitation (Langhorne et al., 2011). However, most of the sample sizes were small, particularly for the study of people with gait ignition failure (*n* = 4) (Vidailhet et al., 1995). There were a few notable findings from these studies. The MRCP peak negativity occurred earlier in older adults compared to younger adults during a cued forward-stepping task, which might reflect an impaired ability to anticipate the timing of the upcoming stimulus (Khanmohammadi et al., 2015) or compensation for delayed force production (Klass et al., 2008). Older adults also had a smaller amplitude of the late MRCP (Fz) during stepping (Khanmohammadi et al., 2015), aligning with findings in simple finger movement tasks (Michalewski et al., 1980; Golob et al., 2005) and suggesting age-related changes in motor planning. People with PD had smaller MRCP amplitudes compared to healthy adults during self-paced ankle dorsiflexion or forward stepping (Vidailhet et al., 1993), but people with PD and freezing of gait had larger MRCP amplitudes compared to healthy adults and people with PD without freezing of gait during a cued button-pressing task (Fearon et al., 2021). These contrasting results may reflect differences between the samples or variability between the tasks. At this stage there is insufficient evidence to draw conclusions from this literature.

Only one study in this review provided information about the characteristics of the MRCP following stroke. This study found no difference in MRCP amplitude or duration during step-ups between the more-affected and less-affected legs (Peters et al., 2018). Thus, knowledge about MRCP characteristics during ecologically valid movement in people with stroke is very limited. More stroke research has been carried out during simple movements, but with variable findings. Fattapposta et al. found that people with acute stroke had a smaller MRCP amplitude during index finger movements when compared to healthy participants (Fattapposta et al., 2008), which might reflect the suppression of cortical excitability in the affected hemisphere that occurs early after stroke (Stinear et al., 2015). Interestingly, in this study the MRCP amplitude increased over the subsequent 12 months (Fattapposta et al., 2008), possibly reflecting motor recovery. Similarly, other chronic stroke studies have shown MRCPs with earlier onsets and larger amplitudes; this has been observed in the more-affected limb compared with a healthy control limb during a horizontal shoulder flexion and elbow extension task (Daly et al., 2006) and during attempted finger flexion/extension in people with stroke who have severe paresis (Yilmaz et al., 2014). This suggests that people with chronic stroke may need greater levels of cortical activation to produce or attempt simple movements of the affected arm. In contrast, other studies of simple finger movements have shown that smaller MRCP amplitudes are maintained in the affected hemisphere in the chronic stage of stroke (Wiese et al., 2005; Dean et al., 2012), which may reflect poor recovery of movement. Studies of simple movements following stroke have also shown variability in the location of MRCP signals (Daly et al., 2006; Yilmaz et al., 2014), which likely reflects cortical reorganization in response to the lesion (Hosp and Luft, 2011). Further research is needed in stroke and other clinical populations. Longitudinal studies across the recovery process would increase our understanding of how the MRCP could be used as a biomarker of recovery. Ideally, ecologically valid movements should be investigated, however, it is acknowledged that simple movement tasks may be more achievable for individuals with more severe impairment.

### Movement-Related Cortical Potentials as Outcome Measures

Five of the six experimental studies utilized the MRCP as an outcome measure; three of these studies detected changes in the MRCP in response to the interventions (Barthel et al., 2001; Wright et al., 2012a; Fromer et al., 2016) and one small study observed changes but did not perform a statistical analysis (Peters et al., 2020). The movement tasks used to record the signal were all relevant to the interventions being assessed (Barthel et al., 2001; Wright et al., 2012a; Fromer et al., 2016; Mizusaki et al., 2019; Peters et al., 2020). For example, Peters et al. used step-ups to record the MRCP, which were also part of the fast muscle training and stepping activation intervention under investigation (Peters et al., 2020). Two other studies trained dart throwing and used a simulated or real dart throw to record the MRCP (Fromer et al., 2016; Mizusaki et al., 2019). While this body of research is small, it is promising that these studies have successfully measured intervention efficacy using an MRCP recorded during ecologically valid movements. However, the ecological validity of these movements could be further improved; for example, in Fromer et al. the dart throw was simulated with a Wii remote rather than using a real dart and dartboard (Fromer et al., 2016). One of the limitations of analyzing MRCPs is that the signal is detected prior to movement, and therefore more suited to measuring discrete tasks (e.g., stepping) rather than continuous tasks (e.g., walking, climbing stairs). This limitation was illustrated in a study where the movement of interest was continuous cycling but the MRCP was recorded during right-leg kick-type movements on a cycle ergometer (Barthel et al., 2001). Given the specificity of neural plasticity, one would not necessarily expect changes in one task to transfer to improvements in another (Kleim and Jones, 2008). One paper in this review did detect an MRCP-like signal during continuous gait (Knaepen et al., 2015). Further research in continuous movements, such as walking, is needed to determine whether the MRCP can be measured in these tasks and to determine the potential of MRCPs to measure changes following walking interventions. Importantly, if the MRCP is to be useful as an outcome measure following rehabilitation interventions, its reliability must be established. Surprisingly, despite its discovery over 50 years ago, the reliability of the MRCP remains untested in both simple and complex movements. Lack of stability in the MRCP signal could explain many of the contrasting findings in the literature and therefore must be a priority for future research.

### Movement-Related Cortical Potential-Driven Interventions

One experimental study in this review utilized the MRCP within a BCI neuromodulatory intervention; this study reported that the MRCP was recorded during a complex reach-to-grasp task, and thus was determined to be ecologically valid, however, there was limited detail about the features of the movement task (Mrachacz-Kersting et al., 2019a). In addition, results from the seven participants in this study were preliminary and no statistical analysis was performed. Multiple other studies have tested the same MRCP-based intervention, but these have been largely limited to simple movements and laboratory environments, with most studies involving healthy participants (Mrachacz-Kersting et al., 2012; Niazi et al., 2012; Kristensen et al., 2013; Jochumsen et al., 2015b, 2016; Mrachacz-Kersting and Aliakbaryhosseinabadi, 2018; Olsen et al., 2018), a few laboratory-based studies involving people with stroke (Mrachacz-Kersting et al., 2016; Olsen et al., 2020), and only one stroke study carried out in a subacute rehabilitation setting (Mrachacz-Kersting et al., 2019b). Expanding the use of this MRCP-based neuromodulatory intervention into ecologically valid movement tasks would increase the likelihood of feasibly translating this intervention into rehabilitation practice, where simple, single-joint movements are unlikely to provide the demands needed to promote recovery (Kleim and Jones, 2008).

Within rehabilitation there is also potential for other MRCP-driven devices. For example, for people with severe motor impairments such as spinal cord injury or amyotrophic lateral sclerosis, the MRCP may be a means for controlling assistive devices such as wheelchairs, robotic arms, or communication tools (Millan et al., 2010). This review did not identify any studies which used the MRCP from an ecologically valid movement as a control signal for an external assistive device. Several studies in this review showed that the MRCP from various ecologically valid movements can be detected and classified in healthy people and people with stroke. However, the MRCPs slow potential makes it difficult to obtain high classification accuracies. The highest classification accuracies are, not surprisingly, obtained when the number of classes is low, with accuracy reducing as more classes are added into the classification. The best discrimination is generally obtained when classifying between a movement and idle/rest activity (Jochumsen and Niazi, 2020b; Schwarz et al., 2020b). One of the challenges of using the MRCP during ecologically valid movement is that it shares the signal bandwidth with motion artifacts and therefore it can be challenging to implement the pre-processing required to remove noise during online classification (Kline et al., 2015; Oliveira et al., 2016; Richer et al., 2020). Indeed, a number of studies in this review excluded participant data for this reason (see Table 3). From the studies reviewed, it is difficult to determine which ecologically valid movement types are easiest to classify due to: (1) similarities in the morphology of the premovement signal, (2) different signal processing techniques used for denoising, feature extraction, and classification, (3) the different number of movement classes, and (4) the high inter-participant variability which is inherent in these types of analyses and exacerbated by the different participants used in each study. In addition, in some studies epochs are rejected (with varying criteria) to make the data set cleaner for classification but gives it less resemblance to a real-world scenario. Given the difficulties obtaining high classification accuracies, using traditional machine learning algorithms to process the MRCP signal from ecologically valid movements may not be ideal for controlling external assistive devices. However, given the aim of many BCI assistive devices is to support individuals with more-severe paralysis, MRCPs recorded during simple attempted movements may be sufficient in the early phase of rehabilitation and may still provide some level of device control. Studies have shown that MRCPs during attempted hand movements can be recorded from individuals with spinal cord injury and have the potential to control an upper limb neuroprosthesis (Müller-Putz et al., 2019; Ofner et al., 2019). If a BCI-controlled device requires EEG signals generated during more complex movements, different techniques for enhancing MRCP control should be explored by improving decoding algorithms or introducing cyclic command menus (Xu et al., 2019). Another limitation of MRCPs is the slower transfer rate; more accurate movement classification may be obtained from BCI control signals such as steady-state evoked potentials or P300, which allow higher information transfer rates (Wolpaw et al., 2002). The disadvantage of these approaches is that the user needs to focus on a screen with flickering icons or characters to elicit the signals for controlling the external devices; this can be exhausting and provides additional challenges when transferring the BCI to an outdoor environment. An additional area for development is the use of BCIs to control intelligent vehicles for individuals left unable to drive following injury or illness; however, again, control signals other than MRCPs could be more useful depending on the amount of automation/intelligence that is built into the vehicle. Thus, while there is potential for MRCP-driven device control, MRCPs may be more suited to intervention paradigms where prediction of a movement task (before onset) is required to exploit Hebbian plasticity (Mrachacz-Kersting et al., 2019b).

### Limitations

As a scoping review, this paper has described the breadth of MRCP literature related to ecologically valid movements but has not attempted to critique the quality of research methods or the validity of the findings. It was beyond the scope of this review to provide an in-depth critique of various surface EEG recording or signal processing methods, however, such methods have been described in Supplementary Table 1. A previous narrative review has summarized different EEG methods for decoding movement intention from MRCPs (Shakeel et al., 2015). A future systematic review could investigate such methods in more detail in a more defined population or movement type. In addition, this review was limited to surface EEG recordings and does not address invasive EEG methods; such methods offer an improved signal to noise ratio but require surgical intervention (Volkova et al., 2019) and thus have limited clinical feasibility. The scope of this review did not include imagined, single-joint, or partial upper limb movements; however, it is acknowledged that these simpler or more constrained movements have often been utilized in studies of clinical populations such as stroke (Daly et al., 2006; Fang et al., 2007) and spinal cord injury (Lopez-Larraz et al., 2016; Trincado-Alonso et al., 2018; Ofner et al., 2019), and that the body of clinical literature is broader than what is presented here. Given the lack of clinical studies utilizing MRCPs during ecologically valid movements, future systematic reviews of clinical populations should incorporate a broader range of movement types but ask a more specific question. For example, a systematic review could explore the use of the MRCP to control assistive devices in neurological populations.

In terms of the limitations of the primary research in this review, the description of the MRCP movement parameters was often poor; this may have resulted in the exclusion of studies that used ecologically valid movements but did not adequately describe them. Movement tasks were often modified due to constraints of the research environment and current EEG technology, thus preventing them from meeting the inclusion criteria for an ecologically valid movement. While the 59 included articles investigated tasks deemed to be ecologically valid, there are still limitations in generalizing these findings to the real world. For example, the findings from Mizusaki et al. in seated dart throwing may not necessarily transfer to standing dart throwing, but may resemble dart throwing in wheelchair users (Mizusaki et al., 2019). In addition, due to the heterogeneity of study protocols and the variable findings, caution should be taken when attempting to generalize the findings to different tasks and populations.

## Conclusion

This scoping review synthesized the research evidence investigating the MRCP in ecologically valid movement tasks. The 59 included studies demonstrated that the MRCP has been investigated across a broad range of functional and complex motor tasks, but largely in healthy participants. MRCP characteristics appear to vary across different movement tasks and participant groups. MRCP amplitudes are larger with movements of greater complexity. In addition, the MRCP is altered when a cognitive or secondary physical task is performed prior to, or during the movement, reflecting changes in the resources available for motor preparation. The small body of literature examining clinical populations focused on walking-related tasks. While some of these studies demonstrated differences in MRCP characteristics in older adults and people with PD, more research is needed in populations with neurological or age-related impairments to clearly establish how the MRCP changes with disease progression and recovery, and to determine how altering the physical or cognitive requirements of the task influences motor preparation processes. The MRCP has potential to be used as a measure of intervention efficacy, as shown in five studies in this review; however, further research is needed to establish the reliability of the MRCP during movement tasks that are functionally relevant to rehabilitation and recovery. There is minimal research exploring MRCP-based neuromodulatory interventions during ecologically valid movement tasks and this is an area for further development. Although the MRCP can be used to classify real-world movements, it is difficult to obtain high classification accuracies, and this likely explains the absence of studies using MRCPs from ecologically valid movements to control BCI-driven robotics or intelligent cars. Further research is needed to address the technical challenges associated with recording MRCPs to ensure future studies can focus on ecological validity. This will facilitate the implementation of this research into rehabilitation practice.

## Author Contributions

DT, NS, IN, MJ, SO, GA, and UR devised the conceptual framework for the wider body of research that encompasses this study. SO and GA designed the study and provided supervision of MW and SC. MW, SC, and SO conducted the database search. SO, GA, MW, and SC conducted manuscript screening, with input from IN. MW, SC, SO, GA, MJ, and UR, extracted and cross-checked data. All the authors contributed to the interpretation of data, provided critical feedback, and helped shape the writing. SO, GA, MW, and SC drafted the manuscript.

## Conflict of Interest

The authors declare that the research was conducted in the absence of any commercial or financial relationships that could be construed as a potential conflict of interest.

## Publisher’s Note

All claims expressed in this article are solely those of the authors and do not necessarily represent those of their affiliated organizations, or those of the publisher, the editors and the reviewers. Any product that may be evaluated in this article, or claim that may be made by its manufacturer, is not guaranteed or endorsed by the publisher.
