# Supplementary material for: Electroencephalographic Recording of the Movement-Related Cortical Potential in Ecologically Valid Movements: A Scoping Review
Source: Front Neurosci. 2021 Sep 28;15:721387. doi: 10.3389/fnins.2021.721387 (PMC8505671; doi:10.3389/fnins.2021.721387)
Supplement: Supplementary file 1 [file Table_1.pdf]

Supplementary Table 1. EEG recording and processing methods

Author	Amplifier	Cap and recording sites	Filters	Pre-processing method	Time- or response-locking	Epoch length
Barthel et al., 2001	Battery-powered amplifier and A/D converter (MediSyst GmbH, Linden, Germany)	17-electrode Electrocap (Electro Cap Co., USA). Fz, Cz and Pz, with Cz as reference (10/20 system).	Not reported	Excluded epochs with artefacts, exclusion criteria not stated	Movement onset determined when force transducer attached to pedal reached 171N	-2s to 1s (3000ms) or -2s to 0.01s (2010ms)
Bayliss et al., 2000	Grass amplifiers (analogue bandwidth of 0.1-100Hz). Sampled at 500 Hz.	Electrodes/cap not described. Fz, Cz, CPz, Pz, P3, P4, 2 EOG channels, with mastoid as reference.	Not reported	Excluded epochs with artefacts, exclusion criteria not stated	Changes of traffic light colors	-0.1s to 1s (1100ms)
Berchicci et al., 2017	Three 32-channel BrainAmp amplifiers (BrainProducts GmbH., Munich, Germany). Sampled at 250 Hz.	64-electrode ActiCap (BrainProducts GmbH, Munich, Germany). Cz, C1, C2, Fp1, Fp2, with mastoid as reference (10/10 system).	Band-pass filter (0.01-80 Hz), low-pass IIR filter (25 Hz, 48dB/octave).	Epochs with gross artifacts excluded by visual inspection, eye-blink artefacts corrected with ICA, excluded epochs with amplitude greater than ± 60 µV.	Movement onset determined from biceps brachii EMG (exceeding 10% of resting activity)	-1.5s to 3s (4500ms)
Berchicci et al., 2020	Two BrainAmp amplifiers. Sampled at 250 Hz	64-electrode ActiCap. Fp1, Fp2, C1, Cz, C2, PO3, POz, PO4<br>With mastoid as reference (10/10 system)	2nd order zero-phase Butterworth band-pass filter (0.01-50Hz). Individual averaged MRCPs were further low-pass filtered (15 Hz).	CAR filter. Eye-movement artefacts corrected with ICA. Excluded EEG with amplitude greater than ± 80 µV.	Movement onset determined from force platform	-2.5s to 2.5s (5000ms)
Bodda et al., 2020	Amplifier not reported. Sampled at 128 Hz.	Commercially available 14 Ag/AgCl electrode device (10/20 system)	20th order FIR band-pass filter (5-60 Hz). Notch filter (50 Hz).		Gait phases (accelerometer sensors)	30 seconds standing and 8 seconds walking (38s)
Boulenger et al., 2008	BrainAmp MR (Brain Products GmbH, Munich, Germany). Sampled at 500 Hz.	32 Ag/AgCl electrode ECI electro-cap (Electro-Cap International, Inc., USA). Cz, FC1, FC2, C1, C2, with FP2 as reference (10/20 system).	Band-pass filter (0.01-30Hz, 48dB/octave)	CAR filter including FP2. EOG correction. Excluded epochs with either EEG or EOG greater than ± 200 µV	Cue to move (S2)	-0.98s to 0.7s (1680ms)
Braquet et al., 2020	Amplifier not reported. Sampled at 512 Hz.	128 electrode Ag/AgCl cap (Waveguard®, ANT Neuro).<br>FCz, Cz, CPz and Pz (10/05 system)	Down-sampled (250 Hz). Low-pass filter (100 Hz) to remove harmonic, high-pass filter (0.5 Hz), notch filter (50 Hz).	Epoch creation, channel removal with 5s flatline criterion, automatic channel rejection using correlation analysis, manual channel rejection, spherical channel interpolation, artefact rejection using artifact subspace reconstruction, CAR filter, artefact rejection using ICA	Time-locked to cue to move or response-locked to anticipatory postural adjustment (determined from force platform and kinematic data).	-1.5s to 1s for target onset (2500ms)<br>or -1.5s to 0.65s for anticipatory postural adjustment (2150ms).
Bulea et al., 2014	BrainAmp Amplifier (Brain Products, Gmbh). Sampled at 1000 Hz.	Active electrode, 64-electrode EEG (Brain Products, GmbH, Morrisville, NC) (10-20 system)	8th order zero-phase Butterworth high-pass filter (0.05Hz), 3rd order zero-phase Butterworth band-pass filter (0.1-4Hz).	Automated artefact rejection using artifact subspace reconstruction, down-sampling to 200 Hz.	Movement onset determined from EMG of lower limb muscles	-1.5s to 0s (1500ms) and +1.5s to -1.5s of next repetition (max 5000ms)

Chaisen et al., 2020	Biosignal amplifier (g.USBamp RESEARCH, g.tec, Austria). Sampled at 1200 Hz.	11 electrodes. FCz, C3, Cz, C4, CP3, CPz, CP4, P3, Pz, P4, and POz (10/20 system). Left earlobe as reference.	2nd order non-causal Butterworth high-pass filter (0.05 Hz). Notch filter (50 Hz). Down-sampled (250 Hz).	Artefact removal with ICA	Movement onset determined by lower limb EMG	-1.5s to 1s (2500ms)
de Oliveira et al., 2012	Not reported	20 gold electrodes (EMSAMED, Rio de Janeiro, Brazil). Cz, C3, C4, with linked mastoid as reference (10-20 system).	2nd order Butterworth low-pass filter (10 Hz)	Excluded epochs with EEG greater than ± 100 µV	Movement onset determined by extensor carpi radialis EMG	-2.6s to 0s (2600ms)
do Nascimento et al., 2005	40-channel Nuamps (Neuro Scan Labs). Sampled at 500 Hz.	24 tin electrodes in a custom cap (Electrocap International). FC1, FC2, CF13, CF1, CFZ1, CFZ2, CF2, CF24, C3, C13, C1, CZ1, CZ, CZ2, C2, C24, C4, CP3, CP1, CPZ1, CPZ, CPZ2, CP2 and CP4, and linked earlobes as reference (Adapted 10/20 system).	Band-pass filter (0.1-50 Hz)	Excluded epochs with EOG greater than ± 100 µV.	Heel-off movement onset determined by force plates	-1.5s to 1s (2500ms)
Eilbeigi et al., 2018a	WAY-EEG-GAL dataset from Luciw et al. (2014). Sampled at 500 Hz.	Acticap. F3, Fz, F4, FC1, FC2, C3, Cz, C4, CP1, CP2 (10/20 system).	2nd order zero-phase non-causal Butterworth band-pass filter (0.05-5 Hz)	GocICA and conventional cICA. Down-sampled (100 Hz).	Hand movement onset and start of object lowering determined from kinematic data	-2.5s to 2.5s (5000ms)
Eilbeigi et al., 2018b	WAY-EEG-GAL dataset from Luciw et al. (2014). Sampled at 500 Hz.	See Eilbeigi et al., 2018a	2nd order non-causal Butterworth band-pass filter (0.05-10 Hz)	GocICA and conventional cICA algorithms. Down-sampled (50 Hz).	Hand movement onset determined from kinetic/kinematic data	-2s to 0s (2000ms)
Euler et al., 2016	ANT 64-channel amplifier (ANT Neuro). Sampled at 1024 Hz.	64-electrode WaveGuard Cap	Down-sampled (512 Hz). Low-pass filter (30 Hz)	CAR filter, removed ocular rifts with a principal components-based algorithm, channel with significant noise interpolated using a splines-based method, excluded epochs with EEG > ±75 µV and changes in EEG greater than ± 60 µV with 100ms window, linear detrending.	Onset of first movement in sequence detected from button push, turn or tap.	-2s to 1s (3000ms)
Fearon et al., 2021	DC amplifiers. Sampled at 2048 Hz.	128-electrode ActiveTwo EEG system (BioSemi)	Down-sampled (512 Hz). Band-pass filter (0.1-30 Hz)	Laplacian transformation to calculate 2nd spatial derivative of current source density. Subtracted left FC4 from right FC3. Excluded epochs with EEG ± 80 µV, SD <0.5 µV, >12 channels with artefacts, or no response 200-1200ms following stimulus. Baseline correction using data from -0.1s to 0s.	Appearance of target stimulus	-0.1s to 0.7s (800ms)
Fromer et al., 2012	Not reported. Sampled at 500 Hz.	65-electrode (Ag/AgCl) cap (Electrode-Cap International, Inc (ECI)). Mainly at midline electrodes; Fz, Cz, Pz.	Low-pass filter (35 Hz)	Adaptive artefact correction method using BESA software for eye movements; excluded epochs using thresholds on amplitude, difference and gradient	Appearance of a word or target on screen (S1), 2s prior to cue to move (S2)	-0.2s to 2.5s (2700ms)
Fromer et al., 2016	BrainAmp amplifier (Brain Products). Sampled at 500 Hz.	64-electrode (Ag/AgCl) cap (ECI Inc.). Cz as reference.	Low-pass filter (40 Hz)	CAR filter, adaptive artefact correction method using BESA software for eye movements; excluded epochs using thresholds for amplitude and gradient.	Refer to Fromer et al., 2012	-0.2s to 2.4s (2600ms)

Jacobs et al., 2011	Not reported. Sampled at 1000 Hz.	Ag/AgCl electrodes. Fz, Cz, C3, C4, and Pz (10/20 system), linked earlobes as reference	Band-pass filter (0.05-60 Hz), low-pass filter (5 Hz).	Excluded epochs with EOG greater than ± 100 µV	Cue to move (S2)	-3s to 4s (7000ms)
Jeong et al., 2017	Not reported. Sampled at 1000 Hz.	Ag/Ag Cl electrodes. Cz, C1, C2, CPz, CP1 and CP2, with FPz as ground and FCz as reference (10/20 system).	2nd order Butterworth digital band-pass filter (0.1-10 Hz) Down-sampled (100 Hz).	CAR and Laplacian applied to Cz, C1, C2 and CPz.	Movement onset determined from EMG activity	-6s to 2s (8000ms) Walking intention state was set from -2s to 0s (2000ms)
Jiang et al., 2015	16-channel gUSBamp EEG amplifier (g.tec). Sampled at 1200 Hz.	ActiveCap (Brainproducts GmbH). Fz, FC1, FC2, C3, Cz, C4, CP1, CP2, Pz, T7, T8 and Fp2 (10/20 system). Nasion as ground and right earlobe as reference.	Notch filter (50 Hz)	Artifacts removed with ICA. Large Laplacian filter with center at Cz.	Movement onset determined from 6-axial force plates identifying mediolateral sway >10%	-3s to 3s (6000ms) -1.5s to 0.5s (1000ms) was used for gait initiation detection
Jochumsen & Niazi, 2020	80-channels g.HIAMP (g.tec). Sampled at 1200 Hz.	64-electrode g.GAMMAcap with active g.SCARABEO electrodes (g.tec).	4th order Butterworth digital band-pass filter (0.1-10 Hz)	Excluded epochs with amplitudes greater than ± 150 µV	Cue to move (S2)	-2s to 0.5s (2500ms)
Jung 1982	Transferred by Mingograph	Vertex electrodes at bilateral pre-central cortex, mastoid as reference.	Not reported	Not reported	Movement onset determined from EMG activity from relevant muscles	5s (5000ms) (Start and end points not reported).
Karimi & Jiang, 2019	32-channel g.Nautilus (g.tec). Sampled at 250 Hz.	32-electrode EEG cap using FP1, F3, Fz, F4, C3, Cz, C4, P3, Pz, and P4 (10/20 system). Right earlobe as reference.	2nd order Butterworth digital band-pass filter (0.05-4 Hz)	Not reported	Cue to move (S2)	-1.5s to 4.5s (6000ms)
Khaliliardali et al., 2012	Portable Bio-semi Active Two system (company not named). Sampled at 2048 Hz.	64 Ag/Agcl electrodes (10/20 system).	Down sampled (256 Hz). Butterworth band-pass filter (0.1-1 Hz).	CAR filter	Cue to move (S2)	-1s to 0s. No-go epoch defined as from -4s to -1s. (4000ms)
Khaliliardali et al., 2015	See Khaliliardali et al., 2012	See Khaliliardali et al., 2012	Down sampled (256 Hz). 4th order Butterworth non-causal IIR band-pass filter (0.1-1Hz)	CAR filter, weighted average filter, moving average filter (window of 25 samples).	See Khaliliardali et al., 2012	See Khaliliardali et al., 2012
Khanmohammadi et al., 2015	Brain Quick System 98 (Micromed, Mogliano Veneto, Italy)	64 Ag-AgCl sintered ring electrodes. Fz, Cz and Pz (10/20 system). Fpz as ground and mastoid as reference.	Band-pass filter (0.02-70 Hz)	EEG epochs with artifacts removed with EEGLAB, criteria not reported.	Auditory warning signal (S1), 2s prior to cue to move (S2).	-0.5s to 2.5s (3000ms)
Knaepen et al., 2015	Not reported. Sampled at 1000 Hz.	32 active electrodes (10/20 system). Fp1/2, F3/4/7/8/z, FC1/2/5/6, T7/8, C3/4/z, TP9/10, CP1/2/5/6, P3/4/7/8/z, PO9/10, O1/2/z	Band-pass filter (0.5-30 Hz), notch filter (50 Hz).	Excluded epochs with amplitude values (a) ≥ ± 100 µV, (b) ≥ 5 SDs of the mean kurtosis, (c) ≥ 5 SDs of the mean probability distribution, (d) drifts ≥ 50 µV/epoch and R-square limit ≤ 0.3 and (e) spectra deviating from the mean by ±50 dB in the 0–2 Hz and by +25 or −100 dB in the 20–30 Hz frequency window. Excluded epochs with visual inspection. Excluded independent components using DIPFIT/EEGLAB software.	Left heel strike, using a force sensing resistor attached to participants left heel.	1000ms (start point not reported)

Koester & Shack, 2016	64-channel amplifier (ANT Neuro). Sampled at 512 Hz.	Ag-AgCl electrode cap (10/20 system) Regions of interest: F5, F3, F1, FC3, FC1, and F6, F4, F2, FC4, FC2, and PO5, PO3 P3, P1, O1, and PO6, PO4, P4, P2, O2	Band-pass filter (0.1–30 Hz)	Re-referenced to the average mastoid activity, epochs excluded with moving window approach (200 ms extension with a threshold ± 50 µV), epochs excluded with visual inspection	Time-locked to movement cue (presentation of word) and response-locked to movement onset (release of start button).	-1.3s to 0s (1300ms) and -1.1s to 0s (1100ms)
Kourtis et al., 2013	BioSemi Active-Two Amplifiers (company not named). Sampled at 512 Hz.	Nylon cap with 64 Ag-AgCl electrodes (10/20 system). Mastoid as reference.	Band-pass filter (0.1-60 Hz)	Eye-movement correction performed with Brain Vision Analyzer, criteria not reported.	Cue about type of movement (S1), 200ms prior to cue to move (S2)	-0.3s to 1.7s (2000ms)
Mann et al., 2011	BIOPAC EEG amplifier (EEG100B), (BIOPAC Systems Inc., Santa Barbara, CA)	Six Ag/AgCl electrodes, Electrode-Cap International, Inc. (ECI, Eaton, OH). Cz, C3, C4, P3, P4, FPz (10/20 system). Linked ear reference.	Low-pass filter (70 Hz)	Not reported	Movement onset determined from extensor carpi ulnaris EMG	Not reported
Martinez-Exposito et al., 2017	Not reported. Sampled at 256 Hz.	32 electrodes (10/10 system). Main focuses were: C3, C1, Cz, C2, C4.	Zero-phase band-pass filter (0.05-5Hz).	CAR filter	Movement onset determined from EMG of relevant muscles, 3D gyroscopes and accelerometers.	-3s to 1s (4000ms)
Mizusaki et al., 2019	Not reported. Sampled at 500 HZ.	Ag/AgCl electrode cap. Cz, C3, C4 (10/20 system).	Low-pass filter (200Hz).	Not reported	Movement onset determined from triceps brachii EMG	-2 to 0.5s (2500ms)
Moinnereau et al., 2019	Active Two BioSemi recording device	64-electrode Cap. C1, Cz, C2, CP1, CPz, CP2 (10/20 system). POz as reference.	Down sampled (256 Hz). 4th order Butterworth band-pass filter (0.1-1Hz).	ICA, empirical mode decomposition.	Change in steering angle	-4s to 4s (8000ms)
Mrachacz-Kersting et al., 2019	g.USBamp amplifier (g.tec, GmbH, Austria). Sampled at 1200 Hz	g.GAMMAcap, Austria. C2, CP1, C3, FC5, F3, CP5, FC1, P3, T7 and FP2 (10/20 system). Reference on the earlobe.	Not reported	Not reported	Cue to move (S2)	-2s to 2s (4000ms)
Nann et al., 2019	Wireless, Portable 8-channel EEG system (LiveAmp, Brain Products GmbH, Gilching, Germany). Sampled at 250 Hz.	ActiCap (Brain Products GmbH). Fz, FC1, FC2, C3, Cz, C4, CP1, CP2 (10/10 system). Reference on the mastoid.	Band-pass filter (0.1-3Hz)	Baseline correction using data from -2.5s to -2s. Excluded epochs with EEG greater than ± 100 µV or drifts before movement onset.	Movement onset determined from accelerometer located on occiput	-2.5 to 0.5s (3000ms)
O'Connor, 1986	Not reported	Vertex electrodes placed at Cz. Right earlobe as reference	Low-pass filter (35 Hz)	Not reported	- Lip movement onset determined by EMG electrodes placed below corner of lower lip. - Tapping movement onset	-8s (end point not reported)
Peters et al., 2018	Direct current full-band EEG system (NEURO PRAX EEG, NeuroConn, Ilmenau, Germany). Sampled at 2000 Hz.	64-electrode Ag-AgCl electrode cap. Cz primary site of interest (10-20 system). Bilateral mastoid reference.	Down sampled (500Hz). Band-pass filter (0.25-500 Hz), notch filter (60 Hz).	ICA. Data rejected based on visual inspection.	Movement onset determined from electro-goniometers attached to lateral knees.	-4s to 4s (8000ms)

Peters et al., 2020	Direct current full-band EEG system (NEURO PRAX EEG, NeuroConn, Ilmenau, Germany).	64-electrode Ag-AgCl electrode cap. Cz primary site of interest (10-20 system). Bilateral mastoid reference.	Down sampled (500Hz). Band-pass filter (0.25-100 Hz), notch filter (60 Hz).	ICA. Data rejected based on visual inspection.	Refer to Peters et al., 2018	-4s to 4s (8000ms)
Rashid et al., 2018	OpenBCI (OpenBCI, New York, USA), sampled at 250 Hz. Nuamps (Compumedics Neuroscan, Dresden, Germany). Sampled at 500 Hz.	Quick-Cap with Ag/AgCl electrodes. Fp1, F3, F4, FC3, FCz, FC4, C3, Cz, C4, CP3, CPz, CP4, P3, and P4 (10/20 system). Right mastoid as reference.	2nd order Butterworth band-pass filter (0.05-40 Hz), notch filter (49-51 Hz). Down-sampled (125 Hz). Further 2nd order Butterworth low-pass filter (5 Hz).	ICA. A small spatial filter across FC3, FCz, FC4, C3, Cz, C4, CP3, CPz, and CP4. Epochs with 125 µV peak-peak amplitudes were rejected. Channel removal or interpolation.	Movement onset determined from tibialis anterior EMG	-3s to 3s (6000ms)
Reiser et al., 2020	Mobile LiveAmp EEG amplifier (Brain Products, GmbH). Sampled at 500 Hz.	30-electrode ActiCap Snap cap with slim electrodes (Brain Products, GmbH). Fz used for MRCP analysis (10/20 system). FCz as reference.	4th order Butterworth band-pass filter (0.5-35Hz).	Excluded channels with kurtosis >8 SDs and amplitude >5 SDs. CAR filter. Baseline correction using data from -0.2s to 0s.	Auditory warning signal about type of task (S1), followed by stimulus 500ms later (spoken numbers indicating response required)	−0.8s to 2.4s relative to the warning signal (3000ms)
Russo et al., 2019	32-channel BrainAmp standard amplifier (BrainProducts GmbH., Munich, Germany). Sampled at 250 Hz.	64-electrode ActiCap with non-polarizable sintered Ag/AgCl electrodes (10/10 system). Mastoid as reference.	Band-pass filter (0.01-80 Hz). Further band-pass filter (0.01-30 Hz), low-pass filter (15 Hz), notch filter (50 Hz).	CAR filter. Removal of noisy channels using topographic interpolation automatic approach. ICA for eye movements. Excluded epochs with EEG greater than ± 80 µV.	Movement onset determined from 3 different triggers: EMG, force plates and stereophotogrammetry.	-2.5s to 2.5s (5000ms)
Sburlea et al., 2015a	TMSi Refa amplifier (TMSi, Enschede, The Netherlands). Sampled at 256 Hz.	30-electrode EEG cap with water-based electrodes (TMSi, Enschede, The Netherlands). F3, Fz, F4, FC1, FC2, C3, Cz, C4, CP1, CP2, with linked ear reference (10-10 system).	2nd order zero-phase shift Butterworth band-pass filter (0.1-1 Hz). Down-sampled (10 Hz).	Artifact removal using joint probability function and ICA.	Movement onset determined from tibialis anterior EMG	-6s to 0 (6000ms)
Sburlea et al., 2015b	See Sburlea et al., 2015a	See Sburlea et al., 2015a	2nd order zero-phase shift Butterworth band-pass filter (0.1-1 Hz). Down-sampled (10 Hz).	ICA	Movement onset determined by release of a footswitch under the right foot	-6s to 0 (6000ms)
Sburlea et al., 2017	See Sburlea et al., 2015a,b	See Sburlea et al., 2015a,b	See Sburlea et al., 2015a,b	See Sburlea et al., 2015a,b	See Sburlea et al., 2015a,b	See Sburlea et al., 2015a,b
Schwarz et al., 2018	Bio signal amplifier (gTec, GmbH, Austria). Sampled at 512 Hz.	61 active electrodes. Positioned over the frontal, parietal and temporal lobes (positions were captured using a ELPOS system by Zebris (Zebris Medical GmbH, Germany). AFz as ground and right earlobe as reference.	4th order zero-phase Butterworth filter (0.3-35 Hz). Down-sampled (16 Hz). Further 4th order zero-phase Butterworth band-pass filter (0.3-3 Hz).	CAR filter. Statistical artefact removal: 1) amplitude threshold (±125 µV), 2) abnormal joint probability, 3) abnormal kurtosis. Channel rejection based on the same criteria.	Movement onset determined from button release	-2s to 3s (5000ms)

Schwarz et al., 2020a	1) g.USBamp (gTec, GmbH, Austria). 2) EEG-Versatile system (Bitbrain, Zaragoza, Spain) 3) EEG-Hero headset (Bitbrain, Spain) All systems sampled at 256 Hz.	1) 58 active gel-based electrodes over frontal, central and parietal areas (g.GAMMA/g.LADYbird, gTec, GmbH, Austria). AFz as ground and right earlobe as reference. 2) 32 water-based electrodes over frontal, central and parietal areas (Bitbrain, Spain). AFz as ground and left earlobe as reference. 3) 11 dry electrodes located at FC3, FCz, FC3, C3, C1, Cz, C2, C4, P3, CPz, and CP4 (10/20 system). Left earlobe as reference and ground.	4th order zero-phase Butterworth band-pass filter (0.3-3 Hz) Resampled (16 Hz).	CAR filter. Applied extended infomax ICA for 1 and 2 only. Manual removal of eye-movement artefacts. Removed trials with: 1) amplitude threshold < ±125 μV), 2) abnormal joint probability, 3) abnormal kurtosis.	Movement onset determined from photodiode or force sensors	-2s to 3s (5000ms)
Schwarz et al., 2020b	See (1) in Schwarz et al. (2020a)	See (1) in Schwarz et al. (2020a). AFF2h as ground and right earlobe as reference.	4th order zero-phase Butterworth band-pass filter (0.3-3 Hz) Resampled (16 Hz).	Subspace subtraction algorithm applied to correct for ocular artefacts. Removed trials with: 1) amplitude threshold < ±125 μV), 2) abnormal joint probability, 3) abnormal kurtosis. Channel rejection based on same criteria.	Movement onset determined by movement of hand off a pressure plate	-2s to 3s (5000ms)
Singh et al., 2016	BIOamplifier (DIGITEX Lab. Co. Ltd., Japan)	Six Ag/AgCl electrodes. F3, Fz, F4, C3, Cz and C4 (10-20 system). Mastoid reference.	Band pass filter (0.05-30 Hz). Low-pass filter (4 Hz).	Manual rejection of artifacts (not reported). Baseline correction using data from -4s to -3s	Movement onset determined from quadriceps EMG and gyroscope located on latissimus dorsi	-4s to 2s (6000ms)
Skrzeba & Vogt, 2018	BrainVision Analyser 2.1.0 (Brain Products GmbH, Gilching, Germany).	32 electrode-Ag/AgCl ActiCap (Brain Products GmbH). p1, Fp2, F7, F3, F1, Fz, F2, F4, F8, FC3, FC1, FC2, FC4, C3, C1,F14 Cz, C2, C4, CP3, CP1, CPz, CP2, CP4, P3, P1, Pz, P2, P4, O1, Oz and O2. AFz as ground and FCz as reference.	Band-pass filter (0.03-10 Hz)	Gratton's standard ocular correction for eye artefacts. Baseline correction using data from -3s to -2.5s.	Movement onset determined from extensor carpi radialis EMG	-3s to 1s (4000ms)
Tomyta et al., 2020	Brain Amp MR and Brain Vision Recorder. Sampled at 1 kHz.	32-electrode Ag–Cl EasyCap (Brain Products) (10–20 system). Oz as reference and FPz as ground.	Band-pass filter (0.1-40 Hz).	Converted to averaged reference, baseline correction using data from -250ms to -150ms, removal of epochs exceeding ±100 μV.	Movement onset determined from tapping onset	-428ms to 428ms (856ms)
Varghese et al., 2016	40-channel digital EEG amplifier (Nuamps, Compumedics Neuroscan, USA). Sampled at 1000 Hz.	Quick-Cap with Ag/AgCl electrodes. Linked mastoid reference.	Band-pass filter (0.05-50 Hz).	ICA. Baseline correction using data from -1.2s to -1s. Manual epoch rejection (not reported).	Response-locked to anticipatory postural adjustment and response-locked to 'foot-off' of lateral step (determined from force platform).	-1.5s to 1.5s (3000ms).
Vidailhet et al., 1993	Not reported	9 electrodes. Vertex (Cz), 4 cm anterior to Cz, 2 cm posterior to Cz and 4 cm lateral to each of these electrodes. Linked ear reference.	Band-pass filter (0.03-300 Hz)	Manual rejection of trials with scalp muscle activity and eye-blink artifacts. Normalization using first 0.5s of recording.	Movement onset determined from tibialis anterior EMG	-3s to 1s
Vidailhet et al., 1995	Not reported	See Vidalilhet et al., 1993	See Vidalilhet et al., 1993	See Vidalilhet et al., 1993	See Vidalilhet et al., 1993	-3s to 1s

Vogt et al., 2017	BrainVision Analyser 2.1.0 (Brain Products GmbH, Gilching, Germany).	32 electrode-Ag/AgCl ActiCAP (actiCAP®, Brain Products GmbH). p1, Fp2, F7, F3, F1, Fz, F2, F4, F8, FC3, FC1, FC2, FC4, C3, C1,F14 Cz, C2, C4, CP3, CP1, CPz, CP2, CP4, P3, P1, Pz, P2, P4, O1, Oz and O2. AFz as ground and FCz as reference.	Band-pass filter (0.03-10 Hz)	Gratton's standard ocular correction for eye artefacts. Baseline correction using data from -2.5s to -2s. Manual artefact rejection. Topographical interpolation of electrodes with impedances exceeding 10kΩ.	Movement onset determined from extensor carpi radialis EMG	-2.5 to 1 (-2.5 to 2 with baseline correction).
Welke et al., 2011	Not reported	64 impedance-optimized electrodes (10/20 system).	Not reported	ICA.	Change in steering angle determined by visual inspection	Not reported
Wright et al., 2012a	NeuroScan Synamps amplifier (Compumedics Neuroscan, Charlotte, NC, USA). Sampled at 1000 Hz.	Six, 6mm diameter Ag/AgCl electrodes secured to scalp using Ten-20 conductive EEG paste (DO Weaver, Aurora, CO, USA). FC3, FCz, FC4, C3, Cz and C4, with ground at FPz and linked mastoids as reference (10-10 system).	Band-pass filter (0-30Hz), offline band-pass filter (0-5 Hz).	Epoch rejection when EOG amplitudes exceeded 50μV. Normalization of the epochs based on data from -2.5s to -2s.	Movement onset determined by pressing first cord of scale (electrode attached to guitar)	-2.5s to 0.5s (3000ms)
Wright et al., 2012b	See Wright et al., 2012a	See Wright et al., 2012a	See Wright et al., 2012a	See Wright et al., 2012a. Manual rejection of artifacts (criteria not reported).	See Wright et al., 2012a	See Wright et al., 2012-a
Yazawa et al., 1997	Not reported. Sampled at 200 Hz.	15 shallow cup Ag/AgCl electrodes F3, F1, Fz, F2, F4, C3, C1, Cz, C2, C4, P3, PI, Pz, P2 and P4. Right earlobe as reference.	Band-pass filter (0.05-60 Hz)	Manual rejection of epochs with artifacts (criteria not reported).	Auditory warning signal (S1), 2s prior cue to move (S2)	-1s to 3.5s
Zaepffel & Brochier, 2012	Refa8 high-density amplifier (ANT). Sampled at 1024 Hz.	62 Ag/AgCl electrode elastic cap (Waveguard Active Shield, Advanced Neuro Technology [ANT], Enschede, the Netherlands). Cz, CPz, C1, C2, C3, C4, FCz, FC1, FC2, FC3, FC4, Pz, P1, P2.	No filtering applied during data acquisition	CAR filter. Rejection of epochs with EOG or eye-blink artifacts based on visual inspection. Baseline correction based on 400ms data prior the cue.	Cue to move (S2)	-3.4s to 0.2s (3600ms).

BESA, brain electromagnetic source analysis software; CAR, common average reference; EOG, electrooculography; GocICA, global optimal constrained ICA; ICA, independent component analysis; IIR, infinite impulse response.
